# Estimating Annual Fluctuations in Malaria Transmission Intensity and in the Use of Malaria Control Interventions in Five Sub-Saharan African Countries

**DOI:** 10.4269/ajtmh.19-0795

**Published:** 2020-09-21

**Authors:** RTS,S Epidemiology EPI-MAL-005 Study Group The RTS,S Epidemiology EPI-MAL-005 study group is composed of (per alphabetical order):, Elisha Adeniji, Kwaku Poku Asante, Owusu Boahen, Guillaume Compaoré, Boubacar Coulibaly, Seyram Kaali, Youssouf Kabore, Mathieu Lamy, John Lusingu, Anangisye Malabeja, Petra Mens, Mattéa Orsini, Lucas Otieno, Walter Otieno, Seth Owusu-Agyei, Janet Oyieko, Jean-Yves Pirçon, Nicolas Praet, François Roman, Ali Sie, Valentine Sing’oei, Sodiomon B. Sirima, Khadime Sylla, Roger Tine, Alfred B. Tiono, Mathilda Tivura, Effua Usuf, Stéphanie Wéry

**Affiliations:** 1 Kintampo Health Research Centre, Ghana Health Service, Kintampo, Ghana;; 2 Centre de Recherche en Santé de Nouna, Nouna, Burkina Faso;; 3 Centre National de Recherche et de Formation sur le Paludisme (CNRFP), Ouagadougou, Burkina Faso;; 4 Aixial c/o GSK, Wavre, Belgium;; 5 National Institute for Medical Research (NIMR), Korogwe, Tanzania;; 6 University of Copenhagen, Copenhagen, Denmark;; 7 Parasitology Unit, Department of Medical Microbiology, Academic Medical Center, Amsterdam University Medical Centers, University of Amsterdam, Amsterdam, The Netherlands;; 8 4Clinics c/o GSK, Wavre, Belgium;; 9 KEMRI - Walter Reed Project, US Army Medical Research Directorate-Kenya, Kombewa, Kenya;; 10 GSK, Wavre, Belgium;; 11 Département de Parasitologie, Centre de Recherche de Keur Socé, Faculté de Médecine, Université Cheikh Anta Diop, Dakar, Senegal;; 12 Medical Research Council Unit, The Gambia at London School of Hygiene and Tropical Medicine, Fajara, The Gambia

## Abstract

RTS,S/AS01_E_ malaria vaccine safety, effectiveness, and impact will be assessed in pre- and post-vaccine introduction studies, comparing the occurrence of malaria cases and adverse events in vaccinated versus unvaccinated children. Because those comparisons may be confounded by potential year-to-year fluctuations in malaria transmission intensity and malaria control intervention usage, the latter should be carefully monitored to adequately adjust the analyses. This observational cross-sectional study is assessing *Plasmodium falciparum* parasite prevalence (*Pf*PR) and malaria control intervention usage over nine annual surveys performed at peak parasite transmission. *Plasmodium falciparum* parasite prevalence was measured by microscopy and nucleic acid amplification test (quantitative PCR) in parallel in all participants, and defined as the proportion of infected participants among participants tested. Results of surveys 1 (S1) and 2 (S2), conducted in five sub-Saharan African countries, including some participating in the Malaria Vaccine Implementation Programme (MVIP), are reported herein; 4,208 and 4,199 children were, respectively, included in the analyses. *Plasmodium falciparum* parasite prevalence estimated using microscopy varied between study sites in both surveys, with the lowest prevalence in Senegalese sites and the highest in Burkina Faso. In sites located in the MVIP areas (Kintampo and Kombewa), *Pf*PR in children aged 6 months to 4 years ranged from 24.8% to 27.3%, depending on the study site and the survey. Overall, 89.5% and 86.4% of children used a bednet in S1 and S2, of whom 68.7% and 77.9% used impregnated bednets. No major difference was observed between the two surveys in terms of *Pf*PR or use of malaria control interventions.

## INTRODUCTION

Substantial investment to expand existing malaria interventions has resulted in a reduction in the global incidence rate of malaria between 2010 and 2017.^1^ However, between 2015 and 2017, after stagnation, a slight upward trend in malaria incidence was observed. Malaria remains a major cause of death worldwide, with approximately 93% of all malaria deaths in 2017 occurring in Africa.^1^ To reach the Global Technical Strategy for Malaria 2016–2030 target of reducing global malaria incidence and mortality rates by at least 90% by 2030,^2^ the need for safe and effective malaria vaccines that prevent disease and death and decrease transmission to enable malaria eradication was endorsed and documented in the WHO Malaria Vaccine Technology Roadmap.^3^

GlaxoSmithKline (GSK) has developed, in partnership with the PATH Malaria Vaccine Initiative, a pre-erythrocytic *Plasmodium falciparum* malaria vaccine, RTS,S/AS01_E_ (GSK™, Wavre, Belgium), for routine immunization of infants and children living in sub-Saharan African (SSA) malaria-endemic countries. In 2015, the European Medicines Agency adopted a positive opinion for the use of the vaccine in children aged 6 weeks to 17 months at the first dose.^4^ In January 2016, the WHO recommended a pilot implementation of RTS,S/AS01_E_ in children as of 5 months of age in three to five epidemiological SSA settings with moderate to high malaria transmission.^5^ In April 2017, the WHO announced the vaccine introduction based on a cluster-randomized design in pilot areas of Ghana, Kenya, and Malawi through the national expanded programs on immunization, in the framework of the Malaria Vaccine Implementation Programme (MVIP).^6^ Today, RTS,S/AS01_E_ is the first vaccine implemented for the prevention of malaria.

To assess vaccine safety, effectiveness, and impact, GSK designed a pre- and a post-vaccine introduction observational study (Clinical Trials.gov identifiers: NCT02374450 and NCT03855995, respectively), allowing comparison of the occurrence of malaria cases and adverse events in vaccinated versus unvaccinated children. In parallel with those studies, the present observational cross-sectional annual study (NCT02251704) is estimating *P. falciparum* parasite prevalence (*Pf*PR) and the use of malaria control interventions during up to nine consecutive years, applying standardized methodologies and multiple diagnostic testing. More specifically, considering the WHO recommendation to operate the MVIP in moderate to high transmission areas of SSA, this study will allow 1) characterizing the malaria transmission intensity (MTI) before RTS,S/AS01_E_ vaccine introduction in different countries/areas, including the ones participating in the MVIP; 2) monitoring overtime fluctuations of MTI and of the use of malaria control interventions before and after vaccine introduction in those areas to adjust the pre- and post-vaccine introduction comparison analyses for potential year-to-year and/or geographical variations.

We present here the results of the first two annual surveys that were conducted before vaccine introduction. On completion of all surveys, the data collected in this study involving approximately 50,000 participants representing multiple sites in various SSA countries will provide a unique perspective on malaria prevalence variations across Africa.

## MATERIALS AND METHODS

### Ethics.

The study was approved by national independent Ethics Committees and local institutional review boards in Burkina Faso (BF), Ghana (GH), Kenya (KE), Senegal (SN), and Tanzania (TZ), and conducted in accordance with the provisions of the International Conference on Harmonisation and Good Clinical Practice guidelines.

### Study population.

Individuals aged 6 months to < 10 years, whose parents or legally acceptable representative had provided informed consent for study participation, were randomly selected each year in each of the study sites (see Study design section) using population listings generated from local Health and Demographic Surveillance Systems (HDSS) and following a stratification by age-group (see the Supplemental Appendix Section 1). Children in care, or actively participating in any trial involving the administration of an investigational malaria vaccine and/or drug, were excluded.

### Study design.

Malaria transmission intensity levels are consensus indicators developed by the Global Malaria Eradication Programme to measure malaria endemicity^7^ using a standardized methodology. There are several methods for estimating MTI, including entomological inoculation rates (EIRs), serological conversion rates (SCRs), and blood parasite prevalence. Although EIR is a standard method, the measure is challenging, and the interpretation and comparability of the setting may be difficult because of vector heterogeneity.^8^ The methodology and interpretation of SCRs to classify the intensity of malaria is still not commonly used. *Plasmodium falciparum* prevalence, despite requiring trained staff for slide reading, provides a standardized and relatively easy to implement method to assess MTI in study sites of varied transmission intensity, and has often been used in previous epidemiological studies.^7,9,10^ Therefore, *Pf*PR was the selected method to assess potential variations in MTI in the present study.

The study is multicentric with study sites corresponding to geographically limited catchment areas located in low, moderate, or high MTI regions of SSA, and having an HDSS in place.

Up to nine annual cross-sectional surveys will be conducted during the malaria peak transmission period in each study site (from mid-September to mid-December in Western African sites and from mid-April to mid-August in Eastern African sites). To optimize the detection of the peak transmission, each study site has been equipped with a weather station to record meteorological data such as rainfall, temperature, and humidity. Surveys were conducted during the course of the rainy season preferably when rains decrease, which should correspond to the period of highest malaria transmission. In this article, results of the first two annual surveys are presented. Survey 1 (S1) and survey 2 (S2) were conducted in seven study sites in five SSA countries: BF (Nouna, Saponé), GH (Kintampo), KE (Kombewa), SN (Keur Socé, Niakhar), and TZ (Korogwe). Sites in GH (Kintampo) and KE (Kombewa) are part of the areas where the RTS,S/AS01_E_ vaccination will be implemented through the national Expanded Programs on Immunization in the framework of the MVIP. It is important to note that this study is also conducted in SSA areas where the RTS,S/AS01_E_ vaccine will not be introduced in the framework of the MVIP because GSK initiated the study before the WHO recommendation.^5,11^

### Data collection.

Demographic details (age and gender), medical history, and information on care-seeking behaviors (hospitalization for malaria within the last 3 months, visits to healthcare provider for fever or malaria treatment in the previous 14 days, anti-malaria therapy received within the last 14 days); malaria control intervention usage (bednets [new {not older than a year}, pierced/torn, impregnated], indoor residual spraying [IRS]); the usage of coils, repellents, and local herbs; and malaria potential risk factors (rural/urban area, house construction materials, use of electricity, and open/closed water source) were recorded for all participants at the time of the survey. Axillary body temperature was measured and recorded during the survey visit.

To assess within-site heterogeneity of *Pf*PR, study areas were mapped by villages using grid referencing and divided into 3–14 segments with a minimum of 10 enrolled individuals per segment. Segments will remain unchanged for the duration of the study.

### Assessment of parasitemia.

Both microscopy and Nucleic Acid Amplification Tests (NAATs) were used in parallel on all participants to assess parasitemia. The latter are expected to be more sensitive and specific, particularly in cases of low parasite density.^12,13^ In brief, a blood sample was collected by finger prick for thin and thick blood films for the microscopy assessment and filter paper blood spots for NAATs. Blood smears were examined by two local independent microscopists, and any discrepancies were settled by a third reader. Parasitemia was measured by the examination of 100 high-powered fields on thick smear to determine the presence of parasites; in the case of a positive result, additional 100 fields were examined to assess the presence of multiple species. *Plasmodium* species and sexual forms were identified on thin blood film. Parasite density was computed as the geometric mean of two readings, counting parasites against 200 white blood cells on thin blood film, assuming 8,000 white blood cells/μL. Parasite density was categorized as low (< 2,500 parasites/µL), medium (2,500–9,999), high (10,000–19,999), or very high (> 20,000). In the case of low density (< 10 parasites against 200 leukocytes), parasite count was conducted against 500 white blood cells. The parasite count technique was replicated to count gametocytes. In parallel with microscopy, asexual and sexual parasitemia was assessed by NAATs using both real-time quantitative PCR (QT-PCR) assay and real-time nucleic acid sequence-based amplification (QT-NASBA) assay. Quantitative PCR allowed detection of asexual and sexual parasites combined, the final output being qualitative (positive or negative) and semi-quantitative (high, medium, low, and negative). QT-NASBA allowed detection of gametocytes, the final output being qualitative (positive or negative). Details for blood slide and NAAT assessment of parasitemia are available in the Supplemental Appendix (Sections 2 and 3, respectively).

If fever (i.e., axillary temperature ≥ 37.5°C) was recorded at the time of the visit or reported to have occurred within 24 hours before the visit, a malaria rapid diagnostic test (RDT) was conducted using blood from the finger prick sampling. If the RDT was positive, then treatment was given according to national guidelines. Moreover, any participant identified as being parasite positive following microscopy was traced to receive treatment according to national guidelines.

### Statistical methods.

The planned sample size was 600 participants per study site and per survey distributed as 400 participants between the ages of 6 months to 4 years and 200 participants between the ages of 5 and 9 years. The sample size was calculated to ensure sufficiently narrow CIs around study site–specific *Pf*PR estimates (with a maximum residual standard error of 0.25).

*Plasmodium falciparum* parasite prevalence and prevalence of gametocytes were estimated as the proportion of participants infected, or carrying gametocytes, respectively, among participants tested. Prevalences were estimated by age and by site. The agreement between the two diagnostic methods used in the framework of this study (parasitemia measured by microscopy versus NAATs) was described using the Cohen’s kappa coefficient and assessed using the Landis and Koch scale.^14^ The prevalence of *Plasmodium* species other than *P. falciparum* was estimated as the proportion of infected participants among participants tested. The within-site heterogeneity between segments was tested using Cochran’s *Q*-test based on inverse variance weights.

Malaria control intervention coverage was estimated as the proportion of users among participants for which this information was available. The care-seeking behaviors (treatment sought for malaria or fever in the 14 days before the visit and hospitalization for malaria in the last 3 months before the visit) were described as the proportion of participants having sought for health care among all participants. In addition, a risk factor analysis for malaria infection (dependent variable: *P. falciparum* parasitemia as measured by microscopy) was conducted using a multivariable logistic regression with study site as cluster and using a backward strategy for the selection of significant explanatory variables, that is, predefined potential risk factors and/or the use of malaria control interventions (Supplemental Appendix Section 4). Age was computed as a continuous variable.

## RESULTS

**Figure 1.**
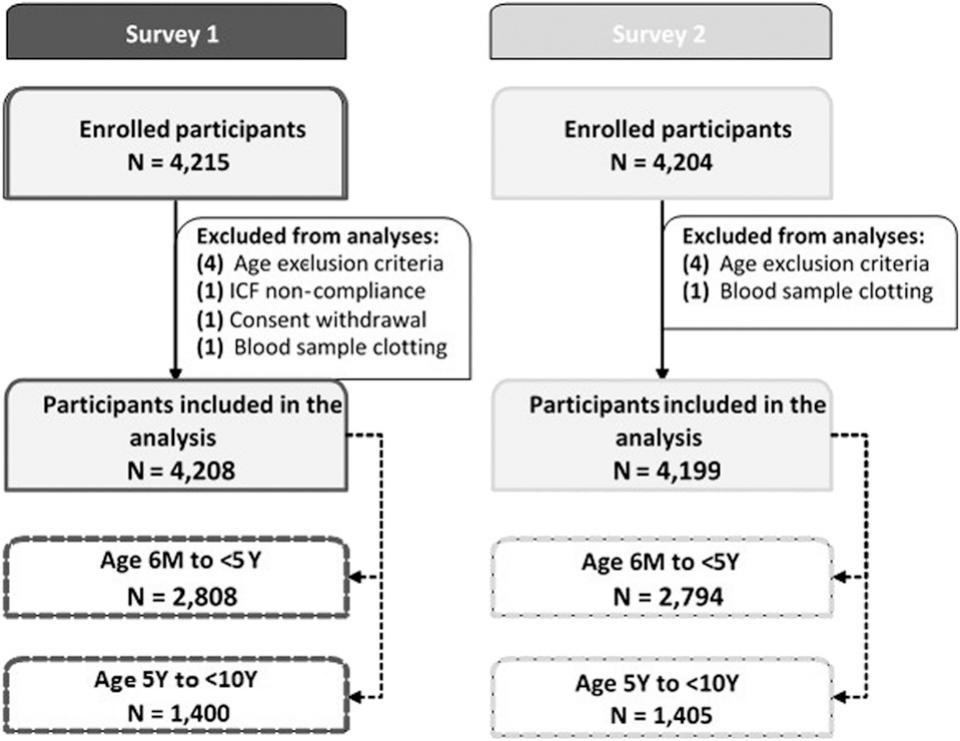
Study design overview and study population included in the analysis. ICF = inform consent form; *N* = number of participants in each group; 6 M to < 5 Y = 6 months to younger than 5 years; 5 Y to < 10 Y = 5 years to younger than 10 years.

Survey 1 and S2 data were collected between October 2014 and August 2015, and between September 2015 and July 2016, respectively. During S1 and S2, 4,215 and 4,204 children were enrolled and 4,208 and 4,199 were included in the analyses (Figure 1), with a balanced distribution across the seven study sites (Supplemental Table 1). Across all sites, 51.3% of participants in S1 and 50.3% in S2 were males (Supplemental Table 2).

### Year-to-year variation in *P. falciparum* prevalence.

**Figure 2.**
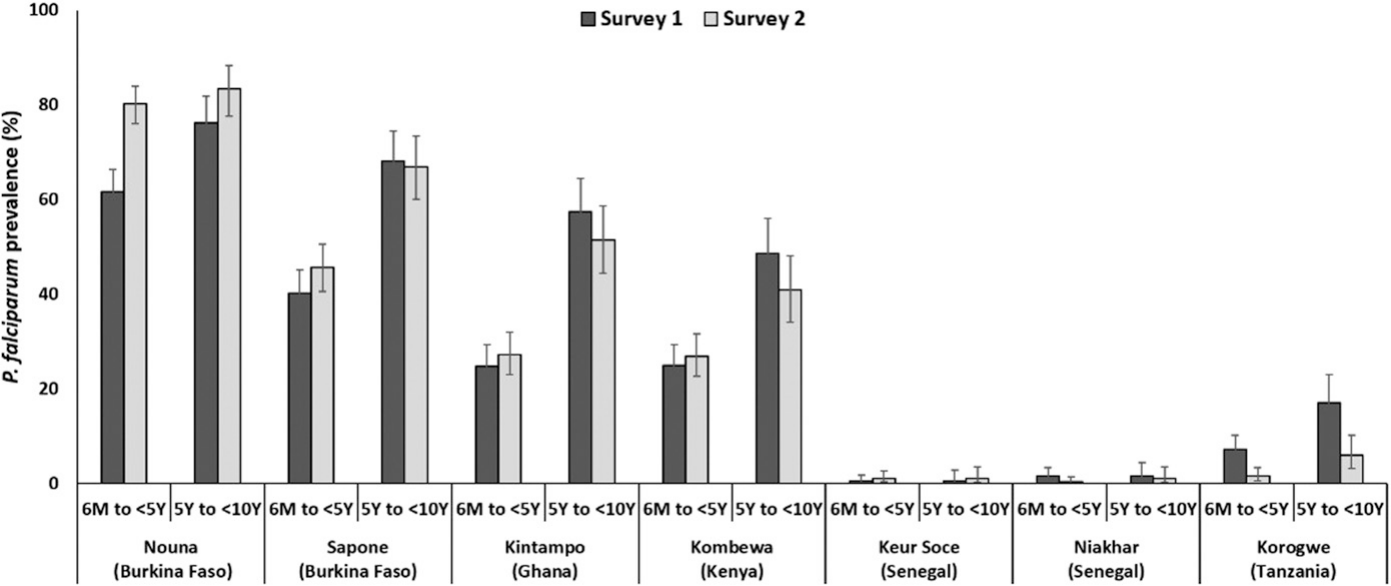
*Plasmodium falciparum* parasitemia prevalence measured by microscopy, by age category, and by site. 6 M to < 5 Y = 6 months to younger than 5 years; 5 Y to < 10 Y = 5 years to younger than 10 years. Error bars depict 95% CI.

*Plasmodium falciparum* parasite prevalence estimated using microscopy varied between study sites in both surveys, with the lowest prevalence figures in Senegalese sites and the highest in BF (Table 1, Figure 2). In Kintampo and Kombewa sites that are located in the MVIP areas, *Pf*PR in children aged 6 months to 4 years ranged from 24.8% to 27.3% depending on the study site and the survey. In both surveys and across all sites except in SN, *Pf*PR was lower in the 6-month to < 5-year than in the 5-year to < 10-year age-group. *Plasmodium falciparum* parasite prevalence was similar in S1 and S2, except for a higher prevalence in S2 in participants from the 6-month to < 5-year age-group in BF Nouna and lower prevalence in S2 in both the 6-month to < 5-year and 5-year to < 10-year age-groups in participants from TZ Korogwe. Significant within-site heterogeneity was detected in all sites, except in SN: BF Nouna (S1 *P* < 0.0001, S2 *P* = 0.0184), BF Saponé (S1 *P* = 0.0321, S2 *P* < 0.0001), TZ Korogwe (S1 *P* = 0.0037, S2 *P* = 0.0275), KE Kombewa (S1 *P* = 0.0220, S2 *P* < 0.0001), and GH Kintampo (S2 only *P* = 0.0065).

**Table 1:** *Plasmodium falciparum* parasitemia prevalence measured by microscopy by study site and survey according to age-group

		*P. falciparum* parasitemia prevalence measured by microscopy slide reading							
Age-group	Study site	Survey 1				Survey 2			
		*n*	*N*	Proportion (%)	95% CI	*n*	*N*	Proportion (%)	95% CI
6 M to < 5 Y	Nouna, BF	249	404	61.6	56.7; 66.4	321	400	80.3	76.0; 84.0
	Saponé, BF	162	403	40.2	35.4; 45.2	182	399	45.6	40.7; 50.6
	Kintampo, GH	99	400	24.8	20.6; 29.3	109	400	27.3	22.9; 31.9
	Kombewa, KE	100	402	24.9	20.7; 29.4	108	401	26.9	22.7; 31.6
	Keur Socé, SN	2	400	0.5	0.1; 1.8	4	397	1.0	0.3; 2.6
	Niakhar, SN	6	398	1.5	0.6; 3.3	1	397	0.3	0.01; 1.4
	Korogwe, TZ	29	401	7.2	4.9; 10.2	6	400	1.5	0.6; 3.2
5 to < 10 Y	Nouna, BF	154	202	76.2	69.8; 81.9	167	200	83.5	77.6; 88.4
	Saponé, BF	137	201	68.2	61.2; 74.5	134	200	67.0	60.0; 73.5
	Kintampo, GH	115	200	57.5	50.3; 64.4	103	200	51.5	44.4; 58.6
	Kombewa, KE	96	197	48.7	41.6; 55.9	81	198	40.9	34.0; 48.1
	Keur Socé, SN	1	200	0.5	0.01; 2.8	2	203	1.0	0.1; 3.5
	Niakhar, SN	3	200	1.5	0.3; 4.3	2	204	1.0	0.1; 3.5
	Korogwe, TZ	34	200	17.0	12.1; 22.9	12	200	6.0	3.1; 10.3

BF = Burkina Faso; GH = Ghana; KE = Kenya; SN = Senegal; TZ = Tanzania; *n* = number of participants positive for *P. falciparum* parasitemia measured by microscopy; *N* = number of participants with known result for *P. falciparum* microscopy; *P. falciparum = Plasmodium falciparum*; 6 M to < 5 Y = 6 months to younger than 5 years; 5 Y to < 10 Y = 5 years to younger than 10 years; 95% CI = exact 95% CI.

Similar trends were observed when measured by QT-PCR, with *Pf*PR varying from 2.7% in SN Niakhar to 69.9% in BF Nouna (not measured in SN Keur Socé) in S1 and from 1.3% in SN Keur Socé to 76.3% in BF Nouna in S2 (Supplemental Table 3).

The prevalence of gametocytes measured by microscopy ranged from 0.5% to 4.0% in S1 and from 0.0% to 5.3% in S2 in all sites, except in BF (15.8% and 22.7% in S1 and 12.8% and 18.2% in S2 in Nouna and Sapone, respectively). Among participants tested positive for asexual parasites, 7.5–66.7% carried gametocytes in S1 (Kintampo and Keur Socé, respectively) and 0.0–83.3% in S2 (Niakhar and Keur Socé, respectively) (Table 2). The proportion of infected participants carrying gametocytes as estimated by QT-NASBA ranged from 13.3% in SN Niakhar to 59.2% in BF Sapone in S1 (not measured in Keur Socé) and from 7.1% in SN Niakhar to 66.7% in SN Keur Socé in S2 (Supplemental Table 3).

**Table 2:** Gametocyte results measured by microscopy according to *P. falciparum* infection status per study site and survey

Study site, *n* (%)	Gametocytes measured by microscopy			
	Participants positive for *P. falciparum* by microscopy		Participants tested for *P. falciparum* by microscopy	
	Survey 1	Survey 2	Survey 1	Survey 2
Nouna, BF	*N* = 403	*N* = 488	*N* = 606	*N* = 600
Positive	89 (22.1)	76 (15.6)	96 (15.8)	77 (12.8)
Saponé, BF	*N* = 299	*N* = 316	*N* = 604	*N* = 599
Positive	111 (37.1)	84 (26.6)	137 (22.7)	109 (18.2)
Kintampo, GH	*N* = 214	*N* = 212	*N* = 600	*N* = 600
Positive	16 (7.5)	23 (10.9)	18 (3.0)	32 (5.3)
Kombewa, KE	*N* = 196	*N* = 189	*N* = 599	*N* = 599
Positive	19 (9.7)	8 (4.2)	24 (4.0)	9 (1.5)
Keur Socé, SN	*N* = 3	*N* = 6	*N* = 600	*N* = 600
Positive	2 (66.7)	5 (83.3)	5 (0.8)	5 (0.8)
Niakhar, SN	*N* = 9	*N* = 3	*N* = 598	*N* = 601
Positive	1 (11.1)	0.0 (0)	3 (0.5)	0.0 (0)
Korogwe, TZ	*N* = 63	*N* = 18	*N* = 601	*N* = 600
Positive	7 (11.1)	1 (5.6)	8 (1.3)	1 (0.2)

BF = Burkina Faso; GH = Ghana; KE = Kenya; SN = Senegal; TZ = Tanzania; *n* (%) = number (percentage) of participants in a given category; *N* = total number of participants; *P. falciparum = Plasmodium falciparum*.

### Agreement between diagnostic tests.

Across surveys, approximately 21.6% of participants with a positive QT-PCR result had a negative microscopy reading (285 of 1,268 positive participants per QT-PCR in S1 and 296 of 1,417 in S2) and around 8.8% of participants positive for microscopy had a negative result with QT-PCR (92 of 1,075 positive participants per microscopy in S1 and 110 of 1,231 in S2) (Table 3). The proportion of participants with a negative result by microscopy among participants with a positive QT-PCR ranged between 42.9% and 78.6% in sites with low MTI (Niakhar, Keur Socé, and Korogwe).

**Table 3:** Agreement between microscopy and QT-PCR test results by survey

Study site	*P. falciparum* measured by microscopy		Survey 1					Survey 2				
			QT-PCR					QT-PCR				
			Positive (*N* = 1,268)		Negative (*N* = 2,069)		Total *N* = 3,337	Positive (*N* = 1,417)		Negative (*N* = 2,737)		Total (*N* = 4,154)
			*n*	%	*n*	%		*n*	%	*n*	%	
Nouna, BF	Positive	*n*	347	90.1	38	9.9	385	400	82.1	87	17.9	487
		%	85.5	–	21.7	–		87.5	–	61.3	–	
	Negative	*n*	59	30.1	137	69.9	196	57	50.9	55	49.1	112
		%	14.5	–	78.3	–		12.5	–	38.7	–	
	Total	*n*	406	–	175	–		457	–	142	–	–
Saponé, BF	Positive	*n*	212	99.1	2	0.9	214	312	98.7	4	1.3	316
		%	86.2	–	1.1	–		80.0	–	1.9	–	
	Negative	*n*	34	16.0	178	84.0	212	78	27.6	205	72.4	283
		%	13.8	–	98.9	–		20.0	–	98.1	–	–
	Total	*n*	246	–	180	–	–	390	–	209	–	–
Kintampo, GH	Positive	*n*	188	90.0	21	10	209	201	94.8	11	5.2	212
		%	81.4	–	6.3	–		71.5		3.4	–	
	Negative	*n*	43	12.1	313	87.9	356	80	20.6	308	79.4	388
		%	18.6	–	93.7	–		28.5		96.6	–	
	Total	*n*	231	–	334	–	–	281	–	319	–	–
Kombewa, KE	Positive	*n*	174	88.8	22	11.2	196	185	97.9	4	2.1	189
		%	66.4	–	6.5	–		78.4	–	1.1	–	
	Negative	*n*	88	21.8	315	78.2	403	51	12.4	359	87.6	410
		%	33.6	–	93.5	–		21.6		98.9	–	
	Total	*n*	262	–	337	–	–	236		363	–	–
Keur Socé, SN	Positive	*n*	–	–	–	–	–	4	66.7	2	33.3	6
		%	–	–	–	–		57.1		0.4	–	
	Negative	*n*	–	–	–	–	–	3	0.5	547	99.5	550
		%	–	–	–	–		42.9	–	99.6	–	
	Total	*n*	–		–	–	–	7	–	549	–	–
Niakhar, SN	Positive	*n*	7	87.5	1	12.5	8	3	100.0	0	0.0	3
		%	46.7	–	0.2			21.4	–	0.0	–	–
	Negative	*n*	8	1.4	549	98.6	557	11	1.8	587	98.2	598
		%	53.3	–	99.8	–		78.6	–	100.0	–	–
	Total	*n*	15	–	550	–	–	14	–	587	–	–
Korogwe, TZ	Positive	*n*	55	87.3	8	12.7	63	16	88.9	2	11.1	18
		%	50.9	–	1.6	–		50.0	–	0.4	–	–
	Negative	*n*	53	9.9	485	90.1	538	16	2.7	566	97.3	582
		%	49.1	–	98.4	–		50.0	–	99.6	–	–
	Total	*n*	108	–	493	–	–	32	–	568	–	–
Overall total	Positive	*n*	983	91.4	92	8.6	1,075	1,121	91.1	110	8.9	1,231
		%	77.5		4.4	–		79.1		4.0	–	
	Negative	*n*	285	12.6	1,977	87.4	2,262	296	10.1	2,627	89.9	2,923
		%	22.5	–	95.6	–		20.9	–	96.0	–	
	Total	*n*	1,268	–	2,069	–	–	1,417	–	2,737	–	–

BF = Burkina Faso; GH = Ghana; KE = Kenya; SN = Senegal; TZ = Tanzania; *n* = number of participants in a given category; *P. falciparum* = *Plasmodium falciparum*; QT-PCR = quantitative PCR; % = percentage of participants with available results.

Across all sites, Cohen’s kappa coefficient between qualitative results of the two methods of measurement of parasitemia (microscopy versus QT-PCR) using the Landis and Koch scale showed a substantial agreement in both surveys (S1: kappa = 0.75 [95% CI: 0.73; 0.78]; S2: kappa = 0.78 [95% CI: 0.75; 0.80]). Cohen’s kappa coefficient between semi-quantitative results for parasitemia measured by microscopy versus QT-PCR also showed a substantial agreement in both S1 (kappa = 0.65 [95% CI: 0.63; 0.68]) and S2 (kappa = 0.66 [95% CI: 0.64; 0.68]).

Between 25.0% (192 of 767 in S2) and 33.8% (185 of 548 in S1) of participants tested positive for gametocytes by QT-NASBA were also detected positive by microscopy (Supplemental Table 4).

### Prevalence of *Plasmodium* species other than *P. falciparum*.

Infection with *Plasmodium malariae* was observed in 1.5% of participants in S1 and 3.0% of participants in S2 (Supplemental Table 5). Coinfection with both *P*. *malariae* and *P*. *falciparum* was more frequent than single infection with *P*. *malariae* alone in both surveys (S1: 4.6% versus 0.2%; S2: 7.4% versus 1.2%). Of the 62 participants in S1 and 128 participants in S2 infected with *P. malariae*, 55 (88.7%) and 91 (71.1%) were also infected with *P. falciparum*, respectively. Infection with *Plasmodium ovale* was low in both S1 and S2 (0.5% and 0.2% of participants). *Plasmodium vivax* was not observed in S1 and in only one participant in S2. Infections with species other than *P. falciparum* were mostly observed in sites of medium-to-high *P. falciparum* prevalence.

### Year-to-year variation in the use of malaria control interventions.

Overall, 89.5% and 86.4% of children used a bednet the night before the survey in S1 and S2, respectively (Table 4, Supplemental Table 6). The highest use of bednets was in KE Kombewa (97.5%) in S1 and TZ Korogwe (99.2%) in S2, and the lowest in GH Kintampo (70.2%) in S1 and SN Niakhar (69.6%) in S2 (Table 4). A decrease in usage between the two surveys was observed in Kombewa, Keur Socé, and Niakhar, whereas an increase was observed in Kintampo and Korogwe.

**Table 4:** Bednet usage the night before the survey by study site and survey

Site, country	Bednet usage the night before the survey			
	Survey 1 (*N* = 4,208)		Survey 2 (*N* = 4,199)	
	*N*	% (95% CI)	*n*	% (95% CI)
Nouna, BF	547	90.3 (87.6; 92.5)	530	88.3 (85.5; 90.8)
Saponé, BF	557	92.2 (89.8; 94.2)	534	89.1 (86.4; 91.5)
Kintampo, GH	421	70.2 (66.3; 73.8)	497	82.8 (79.6; 85.8)
Kombewa, KE	584	97.5 (95.9; 98.6)	534	89.1 (86.4; 91.5)
Keur Socé, SN	568	94.7 (92.6; 96.3)	520	86.7 (83.7; 89.3)
Niakhar, SN	547	91.5 (88.9; 93.6)	418	69.6 (65.7; 73.2)
Korogwe, TZ	544	90.5 (87.9; 92.7)	595	99.2 (98.1; 99.7)
Overall	3,768	89.5 (88.6; 90.5)	3,628	86.4 (85.3; 87.4)

BF = Burkina Faso; GH = Ghana; KE = Kenya; SN = Senegal; TZ = Tanzania; *n* (%) = number (percentage) of children using a bednet the night before the visit in each site; *N* = total number of participants; 95% CI = exact 95% CI.

**Figure 3.**
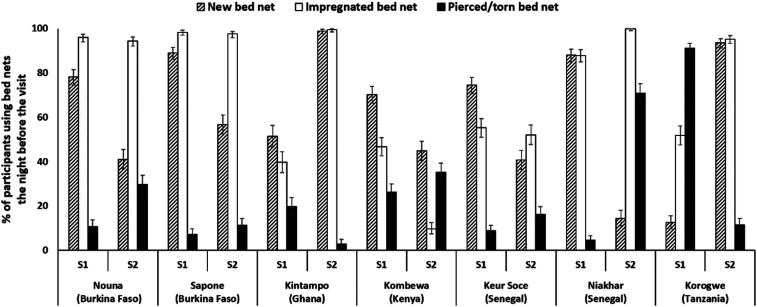
Bednet characterization according to obsolescence, impregnation, and condition, by survey and by site. impregnated bednet = bednet dipped in an insecticide liquid before or after purchase; new bednet = bednet not older than 1 year; S1 = survey 1; S2 = survey 2. Error bars depict 95% CI.

Overall, 70–80% of the bednets were impregnated, 60–70% were new, and approximately 25% were torn (Supplemental Table 6). Details for the characterization of bednet usage (new, impregnated, and pierced/torn) by study site are shown in Figure 3.

Participant’s recall of IRS in the past 12 months was recorded for a very low number of participants (across surveys, 4.1% overall), mainly in SN (Supplemental Table 7).

Overall, usage of coils and repellents was limited, around 10% of the population in both surveys with variations per site between 1% and 40% according to the survey (Supplemental Table 7).

### Year-to-year variation in reported fever and care-seeking behaviors.

Fever in the last 24 hours was reported for approximately a quarter of the participants in both surveys, with differences across study sites ranging from 3.6% in BF Saponé to 63.8% in KE Kombewa in S1 and from 3.0% in SN Keur Socé to 71.6% in KE Kombewa in S2 (Supplemental Table 8). Across all sites, occurrence of fever was higher in *P. falciparum*–infected versus non-infected participants (35.6% versus 21.1% in S1 and 33.5% versus 22.5% in S2, respectively). In both surveys, fever was more frequently reported by participants with higher parasite densities (Supplemental Table 9).

In S1, 15.7% of participants had sought treatment against malaria or fever in the 14 days before the survey compared with 12.8% in S2, ranging from 0.0% to up to 33.3% depending on the study site and on the survey (Table 5, Supplemental Table 10). *Plasmodium falciparum*–infected children sought treatment against malaria or fever more often than non-infected children (20.1% versus 14.0% in S1 and 20.0% versus 9.8% in S2, respectively).

**Table 5:** Care-seeking behaviors (treatment sought for fever and hospitalization for malaria) according to *P. falciparum* infection status by microscopy and survey

Care-seeking behavior	Survey	*P. falciparum* infected			*P. falciparum* not infected			Total		
		*N*	*N*	% (95% CI)	*n*	*N*	% (95% CI)	*n*	*N*	% (95% CI)
Treatment sought for malaria or fever in the previous 14 days	S1	238	1,187	20.1 (17.8; 22.4)	424	3,021	14.0 (12.8; 15.3)	662	4,208	15.7 (14.6; 16.9)
	S2	247	1,232	20.0 (17.8; 22.4)	290	2,967	9.8 (8.7; 10.9)	537	4,199	12.8 (11.8; 13.8)
Hospitalization for malaria in the past 3 months	S1	33	1,187	2.8 (1.9; 3.9)	76	3,021	2.5 (2.0; 3.1)	109	4,208	2.6 (2.1; 3.1)
	S2	42	1,232	3.4 (2.5; 4.6)	77	2,967	2.6 (2.1; 3.2)	119	4,199	2.8 (2.4; 3.4)

*n* (%) = number (percentage) of children in each group; *N* = total number of participants; *P. falciparum = Plasmodium falciparum*; S1 = survey 1; S2 = survey 2; 95% CI = exact 95% CI.

The proportion of participants hospitalized for malaria was 2.6% in S1 and 2.8% in S2, with no marked difference between *P. falciparum*–infected and non-infected participants 2.8% versus 2.5% in S1 and 3.4% versus 2.6% in S2, respectively; (Table 5, Supplemental Table 10**)**.

### Association between potential risk factors and *P*. *falciparum* infection.

An exploratory multivariable model was used to assess the association between potential risk factors or malaria control interventions and *P. falciparum* infection. Across both surveys, houses equipped with electricity (odds ratio [OR]: S1 0.75 [95% CI: 0.61; 0.93]; S2 0.89 [95% CI: 0.80; 0.98]), cement/plaster walls versus mud (OR: S1 0.81 [95% CI: 0.69; 0.96]; S2 0.87 [95% CI: 0.76; 0.99]), and nets on all windows (OR: S1 0.73 [95% CI: 0.63; 0.84]; S2 0.79 [95% CI: 0.66; 0.94]) were associated with a lower risk of infection with *P. falciparum*. In addition, older age was associated with a higher risk of infection (OR: 1.14 [95% CI: 1.05; 1.24] in S1 and 1.09 [95% CI: 1.02; 1.16] in S2; Supplemental Tables 11 and 12).

**Figure 4.**
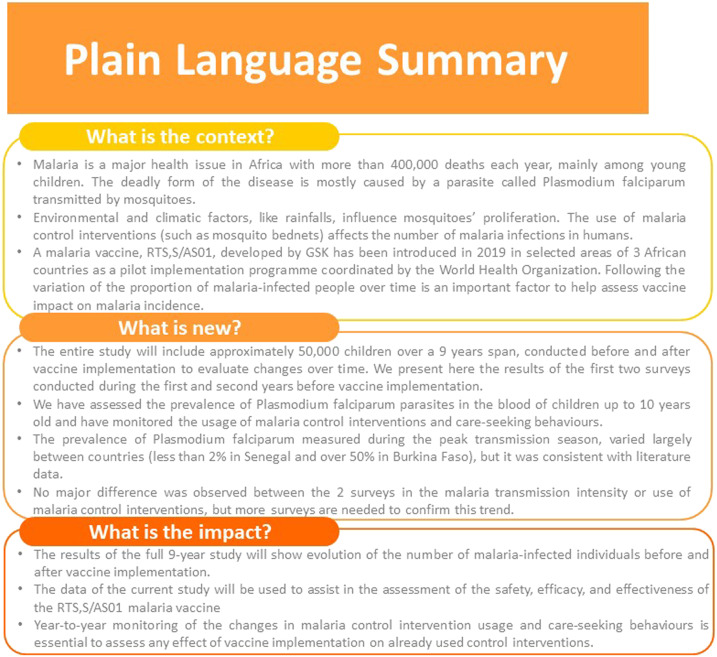
Plain language summary. This figure appears in color at www.ajtmh.org.

Figure 4 represents a plain language summary, which elaborates on the epidemiologic study relevance that could be shared with patients by healthcare professionals.

## DISCUSSION

### Characterizing MTI in different SSA settings.

Using *Pf*PR as a proxy, this study aims at characterizing MTI^15^ in different SSA settings, including areas in GH and KE where the RTS,S/AS01_E_ malaria vaccine is currently introduced in the framework of the MVIP. More specifically, considering the WHO recommendation to operate the MVIP in moderate to high transmission areas of SSA, *Pf*PR in children aged 6 months to 4 years in Kintampo and Kombewa sites was high, ranging from 24.8% to 27.3% depending on the study site and the survey. Similar *Pf*PR was estimated by Drakeley et al.,^9^ indicating a stable mesoendemic MTI level (*Pf*PR = 10–50%) in those areas.^10,16^ *Plasmodium falciparum* parasite prevalence varied largely between sites as it was expected from various preselected transmission intensity areas. In both surveys, the two sites in BF had the highest *Pf*PR and the two sites in SN, the lowest. *Plasmodium falciparum* parasite prevalence rates recorded in this study are in line with previous findings.^9,16–18^

Across most sites, the *Pf*PR was lower in the 6-month to < 5-year than in the 5-year to < 10-year age-group. In addition, a risk factor analysis highlighted an association between older age and higher risk of infection. Those results corroborate the findings of other studies, identifying increasing age as a risk factor for carrying malaria blood stage parasites.^9,16,19^ This may potentially be explained by the fact that younger children benefit from a more focused usage of control interventions (bednets).^20^ Another explanation would be increased immunity in older children due to repeated exposure to the parasite, leading to asymptomatic carriage and a lower probability to be treated than in symptomatic children.^21,22^

Infection with *P. malariae* was observed in a few participants, and infection with *P. ovale* was rare, which supports observations from other studies conducted across SSA.^23–26^ Across all sites and surveys, only one *Plasmodium vivax* infection was identified by microscopy in TZ, which differs from Twohig et al.’s^27^ recent findings of growing evidence of this species in SSA. A high proportion of coinfections with *P*. *falciparum* was observed in *P*. *malariae*–infected participants (between 70% and 90%) and *P*. *ovale*–infected participants (around 60%). Similarly, high percentages of *P. falciparum* coinfection in *P. malariae*–infected participants was previously reported in Guinea (97%),^23^ Uganda (91%),^24^ and in the Democratic Republic of the Congo (90%),^25^ with lower percentages reported in rural BF (67%)^26^ and Benin (34%).^23^

Approximately 20% of participants positive for *P. falciparum* by QT-PCR were undetected by microscopy. Classifying the study sites by MTI, this proportion was higher in low MTI sites. This might be explained by a relatively lower sensitivity of microscopy readings than nucleic acid related techniques, particularly in low parasite density infections, which are more frequently observed in low MTI areas.^12,13^ Nevertheless, the kappa statistic estimated a substantial agreement between microscopy and NAAT techniques using either qualitative or semi-quantitative real-time PCR.

### Monitoring year-to-year variations in MTI and in the use of malaria control interventions.

In addition, the present results are establishing a standardized baseline for further estimation of the year-to-year variations in *Pf*PR and in the use of malaria control interventions, which are key variables to be monitored before and after vaccine introduction in the MVIP areas. Little variation in *P. falciparum* prevalence between the first two annual surveys was observed among most sites. Usage of bednets as a malaria control intervention was high in all sites and in both surveys (ranging 70–99%). Some variation in the usage of bednets was observed in all sites, except in BF.

In S2, participants not using mosquito coils, insecticide sprays, or repellents against malaria vectors were significantly less likely to be infected with *P. falciparum* (OR: S2 0.88 [95% CI: 0.782; 0.988]); however, no such association was observed in S1. Various entomological studies have questioned the efficacy of repellents and coils as effective malaria prevention measures and highlighted the false sense of protection perceived by the user.^28–30^ Our data do not allow to draw robust conclusions at this stage, and the trend observed in S2 should be closely monitored in the subsequent cross-sectional surveys.

### Study limitations.

As with all interview–questionnaire-based studies, this study could have been subject to information or recall bias as data related to bednet usage, control interventions usage, and care-seeking behaviors were collected from parents’ recollection as opposed to objective observation. The impact of these potential information biases on the study results is estimated to be limited because of the proximity in time between the occurrence of the event for which information is collected and the interview itself. Another study limitation may be related to both sensitivity and specificity of microscopy slide readings. Slide reading performances may indeed vary depending on the parasite density and species identification. However, the kappa statistic estimated a substantial agreement between microscopy and QT-PCR, the latter being able to detect a higher number of low density infections than microscopy and RDT.^12,31^ Moreover, this should mainly impact low transmission settings due to the high proportion of low-density infections.

## CONCLUSION

The present article summarizes the results of the first two annual surveys of a larger malaria prevalence study aiming at characterizing MTI in light of the use of malaria control interventions and other environmental factors. Our results confirm that the high *Pf*PR observed in study sites that are part of the MVIP is in line with the WHO recommendation to operate the program in moderate to high transmission areas. In addition, our results are key to inform on the potential occurrence of annual fluctuations in MTI that may influence the assessment of the RTS,S/AS01_E_ vaccine safety, effectiveness, and impact. The observations based on the first two surveys of this study do not indicate major temporal changes in terms of *Plasmodium* prevalence or use of malaria control interventions, but more surveys are needed to confirm this trend. The data generated in this study will be used to create variables to adjust the temporal and concurrent comparison analyses of the RTS,S/AS01_E_ vaccine safety, impact, and effectiveness study. More specifically, the year- and site-specific *Pf*PR computed on unvaccinated study participants will be included as covariates in the regression models of the safety, impact, and effectiveness study to assess annual fluctuations and/or changes due to other malaria control interventions.

To a broader extent, the large sample size of this study (approximately 50,000 participants during the course of 9 years) and the use of a standardized methodology across multiple sites in various countries in SSA using multiple testing will provide a unique perspective on malaria prevalence variations across Africa.

## Supplemental Appendix and tables

Supplemental materials

## References

[1] World Health Organization, 2018 World Malaria Report 2018. Available at: https://www.who.int/malaria/publications/world-malaria-report-2018/report/en/. Accessed March 28, 2019.

[2] World Health Organization, 2015 Global Technical Strategy for Malaria 2016–2030. London, United Kingdom: WHO.

[3] World Health Organization, Malaria Vaccine Funders Group, 2013 Malaria Vaccine Technology Roadmap 2013. Available at: https://www.malariavaccine.org/sites/www.malariavaccine.org/files/content/page/files/TRM_update_nov13.pdf. Accessed February 18, 2019.

[4] European Medicines Agency SMH, 2015 First Malaria Vaccine Receives Positive Scientific Opinion from EMA 2015. Available at: http://www.ema.europa.eu/ema/index.jsp?curl=pages/news_and_events/news/2015/07/news_detail_002376.jsp&mid=WC0b01ac058004d5c1. Accessed March 15, 2018.

[5] World Health Organization, 2018 Malaria vaccine, 2016. WHO position paper–January 2016. Wkly Epidemiol Rec 91: 33–52. 26829826

[6] World Health Organization, 2017 Ghana, Kenya and Malawi to Take Part in WHO Malaria Vaccine Pilot Programme. Available at: https://www.afro.who.int/news/ghana-kenya-and-malawi-take-part-who-malaria-vaccine-pilot-programme. Accessed March 20, 2019.

[7] Hay SI Smith DL Snow RW, 2008 Measuring malaria endemicity from intense to interrupted transmission. Lancet Infect Dis 8: 369–378. 18387849 10.1016/S1473-3099(08)70069-0 PMC2653619

[8] Hay SI Rogers DJ Toomer JF Snow RW, 2000 Annual *Plasmodium falciparum* entomological inoculation rates (EIR) across Africa: literature survey, internet access and review. Trans R Soc Trop Med Hyg 94: 113–127. 10897348 10.1016/s0035-9203(00)90246-3 PMC3204456

[9] Drakeley C 2017 Longitudinal estimation of *Plasmodium falciparum* prevalence in relation to malaria prevention measures in six sub-Saharan African countries. Malar J 16: 433. 29078773 10.1186/s12936-017-2078-3 PMC5658967

[10] Smith DL Guerra CA Snow RW Hay SI, 2007 Standardizing estimates of the *Plasmodium falciparum* parasite rate. Malar J 6: 131. 17894879 10.1186/1475-2875-6-131 PMC2072953

[11] World Health Organization, 2017 Malaria Vaccine Implementation Programme (MVIP). Available at: https://www.who.int/immunization/diseases/malaria/malaria_vaccine_implementation_programme/about/en/. Accessed March 22, 2019.

[12] World Health Organization, 2019 Malaria: Areas of Work – Diagnostic Testing. Available at: https://www.who.int/malaria/areas/diagnosis/nucleic-acid-amplification-tests/en/. Accessed February 18, 2019.

[13] Okell LC Ghani AC Lyons E Drakeley CJ, 2009 Submicroscopic infection in *Plasmodium falciparum*-endemic populations: a systematic review and meta-analysis. J Infect Dis 200: 1509–1517. 19848588 10.1086/644781

[14] Landis JR Koch GG, 1977 The measurement of observer agreement for categorical data. Biometrics 33: 159–174. 843571

[15] The malERA Refresh Consultative Panel on Combination Interventions and Modelling, 2017 malERA: an updated research agenda for combination interventions and modelling in malaria elimination and eradication. PLoS Med 14: e1002453. 10.1371/journal.pmed.1002453 PMC5708628 29190295

[16] Diallo A 2017 An epidemiological study to assess *Plasmodium falciparum* parasite prevalence and malaria control measures in Burkina Faso and Senegal. Malar J 16: 63. 28166794 10.1186/s12936-017-1715-1 PMC5294715

[17] Sylla K 2015 Sero-epidemiological evaluation of *Plasmodium falciparum* malaria in Senegal. Malar J 14: 275. 26173958 10.1186/s12936-015-0789-x PMC4502940

[18] Tine RCK 2013 Parasitic infections among children under five years in Senegal: prevalence and effect on anaemia and nutritional status. ISRN Parasitol 2013: 272701. 10.5402/2013/272701 PMC4890897 27335851

[19] Ceesay SJ 2008 Changes in malaria indices between 1999 and 2007 in The Gambia: a retrospective analysis. Lancet 372: 1545–1554. 18984187 10.1016/S0140-6736(08)61654-2 PMC2607025

[20] Nankabirwa J Brooker SJ Clarke SE Fernando D Gitonga CW Schellenberg D Greenwood B, 2014 Malaria in school-age children in Africa: an increasingly important challenge. Trop Med Int Health 19: 1294–1309. 25145389 10.1111/tmi.12374 PMC4285305

[21] Felger I Maire M Bretscher MT Falk N Tiaden A Sama W Beck H-P Owusu-Agyei S Smith TA, 2012 The dynamics of natural *Plasmodium falciparum* infections. PLoS One 7: e45542. 23029082 10.1371/journal.pone.0045542 PMC3445515

[22] Aron JL, 1983 Dynamics of acquired immunity boosted by exposure to infection. Math Biosci 64: 249–259.

[23] Ceesay SJ 2015 Malaria prevalence among young infants in different transmission settings, Africa. Emerg Infect Dis 21: 1114–1121. 26079062 10.3201/eid2107.142036 PMC4480393

[24] Asua V Tukwasibwe S Conrad M Walakira A Nankabirwa JI Mugenyi L Kamya MR Nsobya SL Rosenthal PJ, 2017 *Plasmodium* species infecting children presenting with malaria in Uganda. Am J Trop Med Hyg 97: 753–757. 28990911 10.4269/ajtmh.17-0345 PMC5590612

[25] Doctor SM 2016 Low prevalence of *Plasmodium malariae* and *Plasmodium ovale* mono-infections among children in the Democratic Republic of the Congo: a population-based, cross-sectional study. Malar J 15: 350. 27392905 10.1186/s12936-016-1409-0 PMC4938993

[26] Gneme A Guelbeogo WM Riehle MM Tiono AB Diarra A Kabre GB Sagnon N Vernick KD, 2013 *Plasmodium* species occurrence, temporal distribution and interaction in a child-aged population in rural Burkina Faso. Malar J 12: 67. 23421809 10.1186/1475-2875-12-67 PMC3583752

[27] Twohig KA Pfeffer DA Baird JK Price RN Zimmerman PA Hay SI Gething PW Battle KE Howes RE, 2019 Growing evidence of *Plasmodium vivax* across malaria-endemic Africa. PLoS Negl Trop Dis 13: e0007140. 30703083 10.1371/journal.pntd.0007140 PMC6372205

[28] Avicor SW Wajidi MFF Owusu EO, 2017 To coil or not to coil: application practices, perception and efficacy of mosquito coils in a malaria-endemic community in Ghana. Environ Sci Pollut Res Int 24: 21138–21145. 28730366 10.1007/s11356-017-9737-3

[29] Hogarh JN Agyekum TP Bempah CK Owusu-Ansah EDJ Avicor SW Awandare GA Fobil JN Obiri-Danso K, 2018 Environmental health risks and benefits of the use of mosquito coils as malaria prevention and control strategy. Malar J 17: 265. 30012143 10.1186/s12936-018-2412-4 PMC6048806

[30] Lukwa N Chiwade T, 2008 Lack of insecticidal effect of mosquito coils containing either metofluthrin or esbiothrin on *Anopheles gambiae* sensu lato mosquitoes. Trop Biomed 25: 191–195. 19287356

[31] World Health Organization, 2017 Malaria Policy Advisory Committee (MPAC) Meeting Report 2017. Geneva, Switzerland: WHO Contract No.: WHO/HTM/GMP/MPAC/2017.21.

