# Supplementary material for: Estimating Annual Fluctuations in Malaria Transmission Intensity and in the Use of Malaria Control Interventions in Five Sub-Saharan African Countries
Source: Am J Trop Med Hyg. 2020 Sep 21;103(5):1883–92. doi: 10.4269/ajtmh.19-0795 (PMC7646796; doi:10.4269/ajtmh.19-0795)
Supplement: Supplementary file 1 [file tpmd190795.SD1.docx]

The following are supplemental files and will be published online only

**Supplemental Appendix**

This appendix has been provided by the authors to give readers additional information about their work.

Appendix“Estimating annual fluctuations in malaria transmission intensity and in the use of malaria control interventions in 5 sub-Saharan African countries”

TABLE OF CONTENTS

Page

[1. SELECTION OF CHILDREN PARTICIPATING IN THE SURVEY 4](#_Toc44679946)

[2. DETERMINATION OF PARASITEMIA BY MICROSCOPY 4](#_Toc44679947)

[3. DETERMINATION OF PARASITEMIA BY nucleic acid amplification test (NAAT) 6](#_Toc44679948)

[4. LIST OF PRE-DEFINED MALARIA CONTROL INTERVENTIONS AND POTENTIAL RISK FACTORS 7](#_Toc44679949)

[5. SUPPLEMENTARY TABLES 9](#_Toc44679950)

[5.1. Study Population and Demographic Characteristics 9](#_Toc44679951)

[5.2. *Plasmodium* parasite prevalence 12](#_Toc44679952)

[5.3. Malaria control interventions 15](#_Toc44679953)

[5.4. Care-seeking behaviours 19](#_Toc44679954)

[5.5. *P. falciparum* infection risk factors analysis 26](#_Toc44679955)

[REFERENCES 28](#_Toc44679956)

LIST OF SUPPLEMENTALTABLES

page

Supplemental Table 1 Number of individuals included in the analysis by study site, age group and survey 9

Supplemental Table 2 Summary of demographic characteristics of individuals by study site and survey 10

Supplemental Table 3 *P. falciparum* results (asexual parasites and gametocytes) by study site and survey measured by NAAT (QT-PCR and QT-NASBA) 12

Supplemental Table 4 Number of individuals carrying gametocytes measured by microscopy compared to gametocytes results measured by QT-NASBA by study site and by survey 13

Supplemental Table 6 Number of individuals having slept under a bednet the night before the visit and characterization of bednets by study site and survey.............. 15

Supplemental Table 7 Number of individuals having used insect repellents or insecticides by study site and survey 17

Supplemental Table 8 Number of individuals presenting with fever reported in the last 24 hours and measured at visit by study site, *P. falciparum* infection status and survey 20

Supplemental Table 9 Number of individuals presenting with fever reported in the last 24 hours and measured at visit by study site, parasite density measured by microscopy and survey 22

Supplemental Table 10 Number of individuals having sought treatment for malaria or fever in the past 14 days and individuals hospitalized for malaria in the last 3 months by study site, *P. falciparum* infection status and survey.............. 25

Supplemental Table 11 Risk factors of being infected with *P. falciparum* (as assessed by microscopy)derived from the fitted logistic regression model with study site as cluster (Survey 1) 27

Supplemental Table 12 Risk factors of being infected with *P. falciparum* (as assessed by microscopy)derived from the fitted logistic regression model with study site as cluster (Survey 2) 28

# SELECTION OF CHILDREN PARTICIPATING IN THE SURVEY

The participant selection process was repeated each year independently meaning that the individuals were different in each cross-sectional survey except if they were re-selected in a subsequent survey by chance. The population listings generated from the demographic surveillance allowed for sampling of the required individuals according to stratification by age group as follows (the number of individuals was approximately plus or minus 5 children):

- 60 children aged 6 months to <1 year
- 120 children aged 1 year
- 120 children aged 2 years
- 50 children aged 3 years
- 50 children aged 4 years
- 40 children aged 5 years
- 40 children aged 6 years
- 40 children aged 7 years
- 40 children aged 8 years
- 40 children aged 9 years.

# DETERMINATION OF PARASITEMIA BY MICROSCOPY

Assessing parasite presence

A 100-field examination of the thick film was conducted to assess presence of parasites and species.[^1^](#_ENREF_1)

*Negative result:* 100 fields free of parasites were to be read before a slide was declared negative.

*Positive result:* If parasites were present within reading of 100 fields, the slide was positive. Positive slides were examined for a further 100 fields to ensure all species present were detected.

Identification of *Plasmodium* species

Positive parasitemia identified on any thick blood film was always identified to species. This was done on thin blood film except in cases of low parasitemia.

Parasite density counting against assumed 8000 leukocytes per microliter

In this method of estimating parasite density, it was assumed that there were 8000 leukocytes per microliter of blood.

- If upon counting 200 leukocytes, 10 or more parasites had been counted, the results were to be recorded as the parasites per 200 leukocytes.
- If upon counting 200 leukocytes, 9 or fewer parasites had been recorded, the reader was to continue counting until 500 leukocytes had been counted and the number of parasites per 500 leukocytes were to be recorded.
- It should be noted that the count was to be by species, and counts for *P. falciparum*were to be made for both gametocytes and asexual parasites.

Criteria for concordance for double reading of slides

All slides were read twice, by two independent readers to quantify the *P. falciparum* parasite presence and density. If slides were judged to be discordant, a third independent read was to be organized in the following cases:

1. The result from one reader was negative and the one of the other was positive.
2. For high and medium positive parasitemia results (blood parasitemia >400/μL), the higher count divided by the lower count was >2.
3. For low parasitemia (blood parasitemia ≤400/μL), the highest reading density was more than one log_10_ higher than the lowest reading.

If the parasitemia result was high or medium in one slide and the result from the other slide reading was low, i.e. one was >400/μL and the other was ≤400/μL, criterion (C) was to be applied.

Determination of final result

If there were two concordant results, the final result was the geometric mean of the two readings.

If the first two readings were discordant then the final result was to follow the following principles:

- For cases of positive/negative discrepancy (A), the majority decision was to be adopted. If the decision was positive, the final result was the geometrical mean of the two positives.
- For cases of three positive reads (B and C), the final result was to be the geometric mean of the two geometrically closest readings.


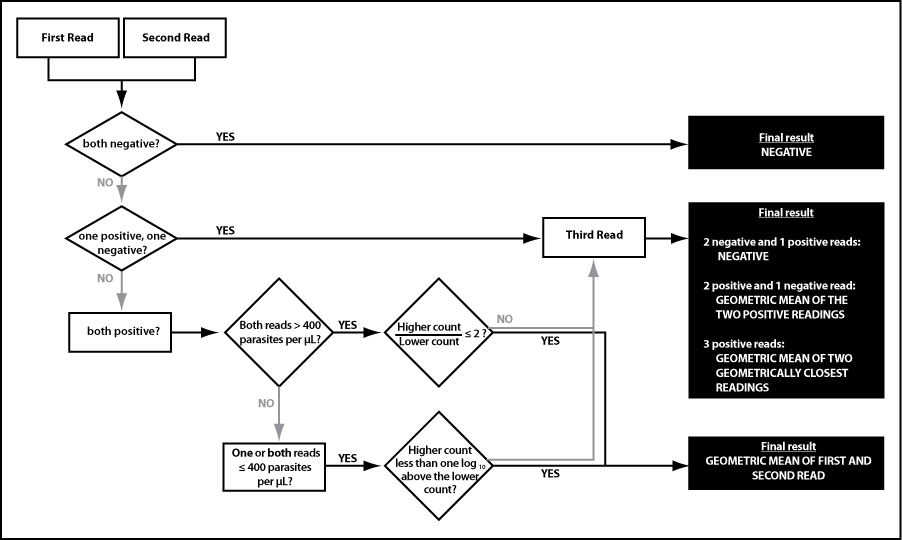


# DETERMINATION OF PARASITEMIA BY nucleic acid amplification test (NAAT)

Determination of parasitemia by nucleic acid amplification test (NAAT) in the study used the following tests:

- quantitative polymerase chain reaction(QT-PCR) for the detection of both DNA (asexual parasites) and RNA (gametocytes);
- quantitative nucleic acid sequence-based amplification (QT-NASBA) for the specific detection of RNA, identifying sexual stages parasites (gametocytes).

Both were performed at AMC (Academic Medical Center, Amsterdam, The Netherlands). Details for QT-PCR are presented in Hermsen et al, 2001[^2^](#_ENREF_2) and for QT-NASBAin Schneider et al, 2004, 2004.[^3^](#_ENREF_3)

# LIST OF PRE-DEFINED MALARIA CONTROL INTERVENTIONS AND POTENTIAL RISK FACTORS

List of malaria control intervention explanatory variables tested in themultivariable logistical regression model:

| Malaria or fever treatment sought for in the past 14 days | Yes vs. No |
| --- | --- |
| Malaria hospitalization in the last 3 months | Yes vs. No |
| Antimalarial or any other medication consumed within 14 days prior to study visit | Yes vs. No |
| Antimalarial drug consumed in the past 14 days | Yes vs. No |
| Other medication consumed over 14 days prior to study visit | Yes vs. No |
| Subject sleep under a bednet last night | Yes vs. No |
| New net (less than 1 year) | No Bednet vs. No |
|  | Yes vs. No |
| Impregnated bednet | Yes vs. No |
|  | No Bednet vs. No |
| Pierced/torn bednet | Yes vs. No |
|  | No Bednet vs. No |
| Number of holes | Less than 5 vs. More or equal to 5 |
|  | No Pierced Bednet vs. More or equal to 5 |
|  | No Bednet vs. More or equal to 5 |
| Use of mosquito coils over 7 days | Yes vs. Missing/No |
| Use of insecticide sprays over 7 days | Yes vs. Missing/No |
| Use of commercial repellents over 7 days | Yes vs. Missing/No |
| Use of traditional repellents over 7 days | Yes vs. Missing/No |
| Use of none of above over 7 days | Yes vs. Missing/No |
| Use of indoor residual spraying (IRS) in the past 12 months to spray interior walls | Yes vs. No |
| Use of indoor residual spraying - number of months ago | >4 vs. 1-2 |
|  | No Residual Spray vs. 1-2 |
|  | 3-4 vs. 1-2 |

IRS = Indoor Residual Spraying, application of a residual insecticide to internal walls and ceilings of housing structures.[^4^](#_ENREF_4)

List of potential risk factor explanatory variables tested in the multivariable logistical regression model:

| Age (in years) | Continuous |
| --- | --- |
| Gender | Male vs. Female |
| Number of persons living in the same part of the house | 4-5 vs. ≤3 |
|  | >5 vs. ≤3 |
| Number of persons enrolled into the study | 1 vs. 2 |
|  | 3 vs. 2 |
|  | >3 vs. 2 |
| Localization | Urban area vs. Rural area |
|  | Semi-Rural Area vs. Rural area |
| Type of location | Town (>10,000 and < 50,000 habitants) vs. Countryside (<10,000 habitants) |
|  | Small city (>50,000 and < 1mil. habitants) vs. Countryside (<10,000 habitants) |
|  | Large city (>1 mil. habitants) vs. Countryside (<10,000 habitants) |
| Main house construction material: Walls | Clay vs. Mud |
|  | Cement/Plaster vs. Mud |
|  | Brick vs. Mud |
|  | Other vs. Mud |
|  | Cement /Paint vs. Mud |
| Main house construction material: Floor | Carpet vs. Natural floor* |
|  | Ceramic tiles vs. Natural floor* |
|  | Parquet/ polished wood vs. Natural floor* |
|  | Clay vs. Natural floor* |
|  | Cement vs. Natural floor* |
|  | Rudimentary floor** vs. Natural floor* |
| Main house construction material: Roof | Tiles vs. Grass/Palm |
|  | Other vs. Grass/Palm |
|  | Iron sheet vs. Grass/Palm |
|  | Clay vs. Grass/Palm |
| Main house construction material: Windows/eaves | Other vs. Open |
|  | No Windows vs. Open |
|  | Closed vs. Open |
|  | Partially open vs. Open |
| Main house construction material: Nets | Nets present on some windows vs. Nets not present |
|  | Nets present on all windows vs. Nets not present |
|  | Other vs. Nets not present |
| Main source of drinking water | Closed water source^†^ vs. Open water source^††^ |
| Is the open source in the compound | No open water vs. No |
|  | Yes vs. No |
| Presence of electricity | Yes vs. No |

* Natural floor = earth, sand, dung.

** Rudimentary floor = wood, palm, bamboo.

^†^ Closed water source (piped water, tube well, dug well, protected well).

^††^ Open water source (unprotected well, spring water, rainwater, tanker truck, surface water).

# SUPPLEMENTAL TABLES

## Study Population and Demographic Characteristics

Supplemental Table 1 Number of individuals included in the analysis by study site, age group and survey

| **Study site** | **6M–<5Y** | | **5–<10Y** | | **All ages** | |
| --- | --- | --- | --- | --- | --- | --- |
|  | **Survey 1** | **Survey 2** | **Survey 1** | **Survey 2** | **Survey 1** | **Survey 2** |
|  | **n** | **n** | **n** | **n** | **n** | **n** |
| **Nouna, BF** | 404 | 400 | 202 | 200 | 606 | 600 |
| **Saponé, BF** | 403 | 399 | 201 | 200 | 604 | 599 |
| **Kintampo, GH** | 400 | 400 | 200 | 200 | 600 | 600 |
| **Kombewa, KE** | 402 | 401 | 197 | 198 | 599 | 599 |
| **KeurSocé, SN** | 400 | 397 | 200 | 203 | 600 | 600 |
| **NiakharSN** | 398 | 397 | 200 | 204 | 598 | 601 |
| **Korogwe, TZ** | 401 | 400 | 200 | 200 | 601 | 600 |
| **All** | 2,808 | 2,794 | 1,400 | 1,405 | 4,208 | 4,199 |

6M–<5Y = Individuals aged 6 months to less than 5 years at informed consent.

5–<10Y = Individuals aged 5 years to less than 10 years at informed consent.

n = number of individuals in a given category.

BF = Burkina Faso; GH = Ghana; KE = Kenya; SN = Senegal; TZ = Tanzania.

Supplemental Table 2 Summary of demographic characteristics of individuals by study siteand survey

| **Study site** | **Characteristics** | | **Survey 1** | **Survey 2** |
| --- | --- | --- | --- | --- |
| **Nouna, BF** | |  | **N=606** | **N=600** |
|  | Age at informed consent (years) | Mean±SD | 3.99±2.69 | 4.00±2.74 |
|  |  | Range | 0.53–9.90 | 0.56–9.97 |
|  | Age group (years) | 6M–<5Y; n (%) | 404 (66.7) | 400 (66.7) |
|  |  | 5–<10Y; n (%) | 202 (33.3) | 200 (33.3) |
|  | Gender | Female; n (%) | 304 (50.2) | 300 (50.0) |
|  |  | Male; n (%) | 302 (49.8) | 300 (50.0) |
| **Saponé, BF** | |  | **N=604** | **N=599** |
|  | Age at informed consent (years) | Mean±SD | 4.05±2.77 | 4.06±2.75 |
|  |  | Range | 0.51–9.93 | 0.50–9.89 |
|  | Age group (years) | 6M–<5Y; n (%) | 403 (66.7) | 399 (66.6) |
|  |  | 5–<10Y; n (%) | 201 (33.3) | 200 (33.4) |
|  | Gender | Female; n (%) | 295 (48.8) | 283 (47.3) |
|  |  | Male; n (%) | 309 (51.2) | 316 (52.8) |
| **Kintampo, GH** | |  | **N=600** | **N=600** |
|  | Age at informed consent (years) | Mean±SD | 3.99±2.74 | 4.02±2.74 |
|  |  | Range | 0.55–9.81 | 0.58–9.94 |
|  | Age group (years) | 6M–<5Y; n (%) | 400 (66.7) | 400 (66.7) |
|  |  | 5–<10Y; n (%) | 200 (33.3) | 200 (33.3) |
|  | Gender | Female; n (%) | 278 (46.3) | 297 (49.5) |
|  |  | Male; n (%) | 322 (53.7) | 303 (50.5) |
| **Kombewa, KE** | |  | **N=599** | **N=599** |
|  | Age at informed consent (years) | Mean±SD | 4.05±2.78 | 4.08±2.75 |
|  |  | Range | 0.57–10.00 | 0.58–9.97 |
|  | Age group (years) | 6M–<5Y; n (%) | 402 (67.1) | 401 (66.9) |
|  |  | 5–<10Y; n (%) | 197 (32.9) | 198 (33.1) |
|  | Gender | Female; n (%) | 279 (46.6) | 315 (52.6) |
|  |  | Male; n (%) | 320 (53.4) | 284 (47.4) |
| **KeurSocé, SN** | |  | **N=600** | **N=600** |
|  | Age at informed consent (years) | Mean±SD | 4.04±2.74 | 4.07±2.76 |
|  |  | Range | 0.55–9.96 | 0.60**–**9.92 |
|  | Age group (years) | 6M–<5Y; n (%) | 400 (66.7) | 397 (66.2) |
|  |  | 5–<10Y; n (%) | 200 (33.3) | 203 (33.8) |
|  | Gender | Female; n (%) | 303 (50.5) | 307 (51.2) |
|  |  | Male; n (%) | 297 (49.5) | 293 (48.8) |
| **Niakhar, SN** | |  | **N=598** | **N=601** |
|  | Age at informed consent (years) | Mean±SD | 4.02±2.77 | 4.08±2.77 |
|  |  | Range | 0.54–9.99 | 0.70–9.83 |
|  | Age group (years) | 6M–<5Y; n (%) | 398 (66.6) | 397 (66.1) |
|  |  | 5–<10Y; n (%) | 200 (33.4) | 204 (33.9) |
|  | Gender | Female; n (%) | 309 (51.7) | 280 (46.6) |
|  |  | Male; n (%) | 289 (48.3) | 321 (53.4) |
| **Korogwe, TZ** | |  | **N=601** | **N=600** |
|  | Age at informed consent (years) | Mean±SD | 4.01±2.75 | 4.03±2.73 |
|  |  | Range | 0.50–9.95 | 0.54–9.97 |
|  | Age group (years) | 6M–<5Y; n (%) | 401 (66.7) | 400 (66.7) |
|  |  | 5–<10Y; n (%) | 200 (33.3) | 200 (33.3) |
|  | Gender | Female; n (%) | 282 (46.9) | 306 (51.0) |
|  |  | Male; n (%) | 319 (53.1) | 294 (49.0) |
| **Overall** |  |  | **N=4,208** | **N=4,199** |
|  | Age at informed consent (years) | Mean±SD | 4.02±2.75 | 4.05±2.75 |
|  |  | Range | 0.50–10.00 | 0.50–9.97 |
|  | Age group (years) | 6M–<5Y; n (%) | 2,808 (66.7) | 2,794 (66.5) |
|  |  | 5–<10Y; n (%) | 1,400 (33.3) | 1,405 (33.5) |
|  | Gender | Female; n (%) | 2,050 (48.7) | 2,088 (49.7) |
|  |  | Male; n (%) | 2,158 (51.3) | 2,111 (50.3) |

N = total number of individuals overall or per site.

n = number of individuals in a given category.

SD = standard deviation.

Range = minimum and maximum values.

6M-<5Y = Individuals aged 6 months to less than 5 years at informed consent.

5-<10Y = Individuals aged 5 yearsto less than 10 years at informed consent.

BF = Burkina Faso; GH = Ghana; KE = Kenya; SN = Senegal; TZ = Tanzania.

## *Plasmodium* parasite prevalence

Supplemental Table 3 *P. falciparum* results (asexual parasites and gametocytes) by study site andsurvey measured by NAAT (QT-PCR and QT-NASBA)

|  | **Survey 1** | | **Survey 2** | |
| --- | --- | --- | --- | --- |
| **Study site** | ***P. falciparum* measured by  QT-PCR** | **Presence of gametocytes measured by  QT-NASBA*** | ***P. falciparum* measured by  QT-PCR** | **Presence of gametocytes measured by  QT-NASBA*** |
| **Nouna, BF;** n (%) | **N=581** | **N’=406** | **N=599** | **N’=456**^†^ |
| Positive | 406 (69.9) | 205 (50.5) | 457 (76.3) | 268 (58.8) |
| Negative | 175 (30.1) | 201 (49.5) | 142 (23.7) | 188 (41.2) |
| **Saponé, BF;** n (%) | **N=426** | **N’=245**^†^ | **N=599** | **N’=390** |
| Positive | 246 (57.7) | 145 (59.2) | 390 (65.1) | 229 (58.7) |
| Negative | 180 (42.3) | 100 (40.8) | 209 (34.9) | 161 (41.3) |
| **Kintampo, GH;** n (%) | **N=565** | **N’=229**^†^ | **N=600** | **N’=281** |
| Positive | 231 (40.9) | 94 (41.0) | 281 (46.8) | 134 (47.7) |
| Negative | 334 (59.1) | 135 (59.0) | 319 (53.2) | 147 (52.3) |
| **Kombewa, KE,** n (%) | **N=599** | **N’=262** | **N=599** | **N’=236** |
| Positive | 262 (43.7) | 81 (30.9) | 236 (39.4) | 120 (50.8) |
| Negative | 337 (56.3) | 181 (69.1) | 363 (60.6) | 116 (49.2) |
| **KeurSocé, SN**;** n (%) | **-** | **-** | **N=556** | **N’=6**^†^ |
| Positive | - | - | 7 (1.3) | 4 (66.7) |
| Negative | - | - | 549 (98.7) | 2 (33.3) |
| **Niakhar, SN;** n (%) | **N=565** | **N’=15** | **N=601** | **N’=14** |
| Positive | 15 (2.7) | 2 (13.3) | 14 (2.3) | 1 (7.1) |
| Negative | 550 (97.3) | 13 (86.7) | 587 (97.7) | 13 (92.9) |
| **Korogwe, TZ;** n (%) | **N=601** | **N’=108** | **N=600** | **N’=32** |
| Positive | 108 (18.0) | 21 (19.4) | 32 (5.3) | 11 (34.4) |
| Negative | 493 (82.0) | 87 (80.6) | 568 (94.7) | 21 (65.6) |

NAAT = Nucleic Acid Amplification Test.

QT-PCR = Quantitative Polymerase Chain Reaction.

QT-NASBA = Quantitative Nucleic Acid Sequence-Based Amplification.

BF = Burkina Faso; GH = Ghana; KE = Kenya; SN = Senegal; TZ = Tanzania.

N = number of individuals tested by QT-PCR for detection of*P. falciparum DNA and/or RNA* per site.

N’ = number of individuals tested positive by QT-PCR being tested by QT-NASBA for detection of *P. falciparum* gametocytes RNA per site.

n = number of individuals in a given category.

*Test for detection of gametocytes, QT-NASBA (RNA specific), was only performed on samples positive by QT-PCR (detecting both DNA and RNA).

**KeurSocé, SN had no valid NAAT results in Survey 1.

^†^ Only individuals with available results are included in N or N’.

Supplemental Table 4 Number of individuals carrying gametocytes measured by microscopy compared to gametocytes results measured by QT-NASBA by study site and by survey

| **Presence of gametocytes measured by microscopy, n (%)** | **Presence of Gametocytes**  **(measured by QT-NASBA)** | | | | **Total** | |
| --- | --- | --- | --- | --- | --- | --- |
|  | **Positive** | | **Negative** | |  |  |
| **Study site** | **Survey 1** | **Survey 2** | **Survey 1** | **Survey 2** | **Survey 1** | **Survey 2** |
| **Nouna, BF** | **N=205** | **N=268** | **N=201** | **N=188** | **N=406** | **N=456** |
|  | 66 (32.2) | 69 (25.7) | 20 (10.0) | 7 (3.7) | 86 (21.2) | 76 (16.7) |
| **Saponé, BF** | **N=145** | **N=229** | **N=100** | **N=161** | **N=245** | **N=390** |
|  | 81 (55.9) | 89 (38.9) | 15 (15.0) | 16 (9.9) | 96 (39.2) | 105 (26.9) |
| **Kintampo, GH** | **N=94** | **N=134** | **N=135** | **N=147** | **N=229** | **N=281** |
|  | 12 (12.8) | 24 (17.9) | 1 (0.7) | 5 (3.4) | 13 (5.7) | 29 (10.3) |
| **Kombewa, KE** | **N=81** | **N=120** | **N=181** | **N=116** | **N=262** | **N=236** |
|  | 18 (22.2) | 7 (5.8) | 4 (2.2) | 2 (1.7) | 22 (8.4) | 9 (3.8) |
| **KeurSocé, SN*** | **-** | **N=4** | **-** | **N=2** | **-** | **N=6** |
|  | - | 3 (75.0) | - | 0 | - | 3 (50.0) |
| **Niakhar, SN** | **N=2** | **N=1** | **N=13** | **N=13** | **N=15** | **N=14** |
|  | 1 (50.0) | 0 | 1 (7.7) | 0 | 2 (13.3) | 0 |
| **Korogwe, TZ** | **N=21** | **N=11** | **N=87** | **N=21** | **N=108** | **N=32** |
|  | 7 (33.3) | 0 | 1 (1.1) | 1 (4.8) | 8 (7.4) | 1 (3.1) |
| **Overall** | **N=548** | **N=767** | **N=717** | **N=648** | **N=1,265** | **N=1,415** |
|  | 185 (33.8) | 192 (25.0) | 42 (5.9) | 31 (4.8) | 227 (17.9) | 223 (15.8) |

QT-PCR = Quantitative Polymerase Chain Reaction.

QT-NASBA = Quantitative Nucleic Acid Sequence-Based Amplification.

N = number of individuals with positive parasitemia as measured by QT-PCR in the given category.

n = number of gametocyte positive individuals measured by microscopy in the given category.

*KeurSocé, SN had no valid QT-PCR results in Survey 1.

BF = Burkina Faso; GH = Ghana; KE = Kenya; SN = Senegal; TZ = Tanzania.

**Supplemental Table 5 Prevalence of *Plasmodium species*other than *P. falciparum* measured by microscopy by study site, *P. falciparum* infection status and survey**

| **Study site** | ***Plasmodium*parasitemia; n (%)** | ***Pf* infected** | | ***Pf* not infected** | | **Total** | |
| --- | --- | --- | --- | --- | --- | --- | --- |
|  |  | **Survey 1** | **Survey 2** | **Survey 1** | **Survey 2** | **Survey 1** | **Survey 2** |
| **Nouna, BF** |  | **N=403** | **N=488** | **N=203** | **N=112** | **N=606** | **N=600** |
|  | *P. malariae* | 2 (0.5) | 30 (6.1) | 2 (1.0) | 27 (24.1) | 4 (0.7) | 57 (9.5) |
|  | *P. vivax* | 0 | 0 | 0 | 0 | 0 | 0 |
|  | *P. ovale* | 1 (0.2) | 0 | 2 (1.0) | 0 | 3 (0.5) | 0 |
| **Saponé, BF** |  | **N=299** | **N=316** | **N=305** | **N=283** | **N=604** | **N=599** |
|  | *P. malariae* | 28 (9.4) | 29 (9.2) | 3 (1.0) | 6 (2.1) | 31 (5.1) | 35 (5.8) |
|  | *P. vivax* | 0 | 0 | 0 | 0 | 0 | 0 |
|  | *P. ovale* | 1 (0.3) | 0 | 0 | 0 | 1 (0.2) | 0 |
| **Kintampo, GH** |  | **N=214** | **N=212** | **N=386** | **N=388** | **N=600** | **N=600** |
|  | *P. malariae* | 9 (4.2) | 15 (7.1) | 2 (0.5) | 3 (0.8) | 11 (1.8) | 18 (3.0) |
|  | *P. vivax* | 0 | 0 | 0 | 0 | 0 | 0 |
|  | *P. ovale* | 3 (1.4) | 1 (0.5) | 5 (1.3) | 1 (0.3) | 8 (1.3) | 2 (0.3) |
| **Kombewa, KE** |  | **N=196** | **N=189** | **N=403** | **N=410** | **N=599** | **N=599** |
|  | *P. malariae* | 16 (8.2) | 16 (8.5) | 0 | 1 (0.2) | 16 (2.7) | 17 (2.8) |
|  | *P. vivax* | 0 | 0 | 0 | 0 | 0 | 0 |
|  | *P. ovale* | 8 (4.1) | 4 (2.1) | 0 | 3 (0.7) | 8 (1.3) | 7 (1.2) |
| **KeurSocé, SN** | | **N=3** | **N=6** | **N=597** | **N=594** | **N=600** | **N=600** |
|  | *P. malariae* | 0 | 0 | 0 | 0 | 0 | 0 |
|  | *P. vivax* | 0 | 0 | 0 | 0 | 0 | 0 |
|  | *P. ovale* | 0 | 0 | 0 | 0 | 0 | 0 |
| **Niakhar, SN** |  | **N=9** | **N=3** | **N=589** | **N=598** | **N=598** | **N=601** |
|  | *P. malariae* | 0 | 0 | 0 | 0 | 0 | 0 |
|  | *P. vivax* | 0 | 0 | 0 | 0 | 0 | 0 |
|  | *P. ovale* | 0 | 0 | 0 | 0 | 0 | 0 |
| **Korogwe, TZ** |  | **N=63** | **N=18** | **N=538** | **N=582** | **N=601** | **N=600** |
|  | *P. malariae* | 0 | 1 (5.6) | 0 | 0 | 0 | 1 (0.2) |
|  | *P. vivax* | 0 | 1 (5.6) | 0 | 0 | 0 | 1 (0.2) |
|  | *P. ovale* | 0 | 1 (5.6) | 3 (0.6) | 0 | 3 (0.5) | 1 (0.2) |
| **Overall** |  | **N=1,187** | **N=1,232** | **N=3,021** | **N=2,967** | **N=4,208** | **N=4,199** |
|  | *P. malariae* | 55 (4.6) | 91 (7.4) | 7 (0.2) | 37 (1.2) | 62 (1.5) | 128 (3.0) |
|  | *P. vivax* | 0 | 1 (0.1) | 0 | 0 | 0 | 1 (0.02) |
|  | *P. ovale* | 13 (1.1) | 6 (0.5) | 10 (0.3) | 4 (0.1) | 23 (0.5) | 10 (0.2) |

*Pf* infected = Individualsinfected with *P. falciparum*parasitemia measured by microscopy.

*Pf* not infected = Individualsnot infected with *P. falciparum*parasitemia measured by microscopy.

N = total number of individuals overall or per site.

n = number of individuals in a given category.

BF = Burkina Faso; GH = Ghana; KE = Kenya; SN = Senegal; TZ = Tanzania.

## Malaria control interventions

Supplemental Table 6 Number of individuals having slept under a bednet the night before the visit and characterization of bednets by study site and survey

| **Study site** | | | **Survey 1** | **Survey 2** |
| --- | --- | --- | --- | --- |
| **Nouna, BF** | |  | **N=606** | **N=600** |
| Participant slept under a bednet the night before the visit | | n | 547 | 530 |
|  |  | % (95% CI) | 90.3 (87.6;92.5) | 88.3 (85.5;90.8) |
|  | New bednet (less than 1 year)* | n | 428 | 218 |
|  |  | % (95% CI) | 78.2 (74.5;81.6) | 41.1 (36.9;45.5) |
|  | Impregnated^†^bednet* | n | 525 | 501 |
|  |  | % (95% CI) | 96.0 (94.0;97.5) | 94.5 (92.2;96.3) |
|  | Pierced/torn bednet* | n | 59 | 158 |
|  |  | % (95% CI) | 10.8 (8.3;13.7) | 29.8 (25.9;33.9) |
| **Saponé, BF** | |  | **N=604** | **N=599** |
| Participant slept under a bednet the night before the visit | | n | 557 | 534 |
|  |  | % (95% CI) | 92.2 (89.8;94.2) | 89.1 (86.4;91.5) |
|  | New bednet (less than 1 year)* | n | 496 | 303 |
|  |  | % (95% CI) | 89.0 (86.2;91.5) | 56.7 (52.4;61.0) |
|  | Impregnated^†^bednet* | n | 548 | 521 |
|  |  | % (95% CI) | 98.4 (97.0;99.3) | 97.6 (95.9;98.7) |
|  | Pierced/torn bednet* | n | 40 | 61 |
|  |  | % (95% CI) | 7.2 (5.2;9.7) | 11.4 (8.9;14.4) |
| **Kintampo, GH** | |  | **N=600** | **N=600** |
| Participant slept under a bednet the night before the visit | | n | 421 | 497 |
|  |  | % (95% CI) | 70.2 (66.3;73.8) | 82.8 (79.6;85.8) |
|  | New bednet (less than 1 year)* | n | 217 | 492 |
|  |  | % (95% CI) | 51.5 (46.7;56.4) | 99.0 (97.7;99.7) |
|  | Impregnated^†^bednet* | n | 167 | 495 |
|  |  | % (95% CI) | 39.7 (35.0;44.5) | 99.6 (98.6;100) |
|  | Pierced/torn bednet* | n | 83 | 15 |
|  |  | % (95% CI) | 19.7 (16.0;23.8) | 3.0 (1.7;4.9) |
| **Kombewa, KE** | |  | **N=599** | **N=599** |
| Participant slept under a bednet the night before the visit | | n | 584 | 534 |
|  |  | % (95% CI) | 97.5 (95.9;98.6) | 89.1 (86.4;91.5) |
|  | New bednet (less than 1 year)* | n | 410 | 239 |
|  |  | % (95% CI) | 70.2 (66.3;73.9) | 44.8 (40.5;49.1) |
|  | Impregnated^†^bednet* | n | 273 | 52 |
|  |  | % (95% CI) | 46.7 (42.6;50.9) | 9.7 (7.4;12.6) |
|  | Pierced/torn bednet* | n | 153 | 188 |
|  |  | % (95% CI) | 26.2 (22.7;30.0) | 35.2 (31.2;39.4) |
| **KeurSocé, SN** | |  | **N=600** | **N=600** |
| Participant slept under a bednet the night before the visit | | n | 568 | 520 |
|  |  | % (95% CI) | 94.7 (92.6;96.3) | 86.7 (83.7;89.3) |
|  | New bednet (less than 1 year)* | n | 423 | 212 |
|  |  | % (95% CI) | 74.5 (70.7;78.0) | 40.8 (36.5;45.1) |
|  | Impregnated^†^bednet* | n | 314 | 271 |
|  |  | % (95% CI) | 55.3 (51.1;59.4) | 52.1 (47.7;56.5) |
|  | Pierced/torn bednet* | n | 50 | 85 |
|  |  | % (95% CI) | 8.8 (6.6;11.4) | 16.3 (13.3;19.8) |
| **Niakhar, SN** | |  | **N=598** | **N=601** |
| Participant slept under a bednet the night before the visit | | n | 547 | 418 |
|  |  | % (95% CI) | 91.5 (88.9;93.6) | 69.6 (65.7;73.2) |
|  | New bednet (less than 1 year)* | n | 482 | 60 |
|  |  | % (95% CI) | 88.1 (85.1;90.7) | 14.4 (11.1;18.1) |
|  | Impregnated^†^bednet* | n | 481 | 418 |
|  |  | % (95% CI) | 87.9 (84.9;90.5) | 100 (99.1;100) |
|  | Pierced/torn bednet* | n | 25 | 296 |
|  |  | % (95% CI) | 4.6 (3.0;6.7) | 70.8 (66.2;75.1) |
| **Korogwe, TZ** | |  | **N=601** | **N=600** |
| Participant slept under a bednet the night before the visit | | n | 544 | 595 |
|  |  | % (95% CI) | 90.5 (87.9;92.7) | 99.2 (98.1;99.7) |
|  | New bednet (less than 1 year)* | n | 68 | 557 |
|  |  | % (95% CI) | 12.5 (9.8;15.6) | 93.6 (91.3;95.4) |
|  | Impregnated^†^bednet* | n | 282 | 567 |
|  |  | % (95% CI) | 51.8 (47.5;56.1) | 95.3 (93.3;96.9) |
|  | Pierced/torn bednet* | n | 496 | 69 |
|  |  | % (95% CI) | 91.2 (88.5;93.4) | 11.6 (9.1;14.4) |
| **Overall** | |  | **N=4,208** | **N=4,199** |
| Participant slept under a bednet the night before the visit | | n | 3,768 | 3,628 |
|  |  | % (95% CI) | 89.5 (88.6;90.5) | 86.4 (85.3;87.4) |
|  | New bednet (less than 1 year)* | n | 2,524 | 2,081 |
|  |  | % (95% CI) | 67.0 (65.5;68.5) | 57.4 (55.7;59.0) |
|  | Impregnated^†^bednet* | n | 2,590 | 2,825 |
|  |  | % (95% CI) | 68.7 (67.2;70.2) | 77.9 (76.5;79.2) |
|  | Pierced/torn bednet* | n | 906 | 872 |
|  |  | % (95% CI) | 24.0 (22.7;25.4) | 24.0 (22.7;25.5) |

95% CI = Exact 95% confidence interval.

N = total number of individuals overall or per site.

n = number of individuals in a given category.

*Denominator = number of individuals who slept under a bednet the night before the visit.

^†^Dipped in liquid insecticidebefore or after purchase.

BF = Burkina Faso; GH = Ghana; KE = Kenya; SN = Senegal; TZ = Tanzania.

Supplemental Table 7 Number of individuals having used insect repellents or insecticides by study site and survey

| **Study site** |  | **Total** | |
| --- | --- | --- | --- |
|  |  | **Survey 1** | **Survey 2** |
| **Nouna, BF** |  | **N=606** | **N=600** |
| Use of mosquito coils over 7 days | n | 109 | 211 |
|  | % (95% CI) | 18.0 (15.0;21.3) | 35.2 (31.3;39.1) |
| Use of insecticide sprays over 7 days | n | 14 | 14 |
|  | % (95% CI) | 2.3 (1.3;3.8) | 2.3 (1.3;3.9) |
| Use of commercial repellents over 7 days | n | 9 | 14 |
|  | % (95% CI) | 1.5 (0.7;2.8) | 2.3 (1.3;3.9) |
| Use of traditional repellents over 7 days | n | 1 | 2 |
|  | % (95% CI) | 0.2 (0.0;0.9) | 0.3 (0.0;1.2) |
| Use of none of the above over 7 days | n | 473 | 364 |
|  | % (95% CI) | 78.1 (74.5;81.3) | 60.7 (56.6;64.6) |
| Use of IRS in past 12 months | n | 0 | 14 |
|  | % (95% CI) | 0 (0.0;0.6) | 2.3 (1.3;3.9) |
| **Saponé, BF** |  | **N=604** | **N=599** |
| Use of mosquito coils over 7 days | n | 1 | 16 |
|  | % (95% CI) | 0.2 (0.0;0.9) | 2.7 (1.5;4.3) |
| Use of insecticide sprays over 7 days | n | 0 | 3 |
|  | % (95% CI) | 0 (0.0;0.6) | 0.5 (0.1;1.5) |
| Use of commercial repellents over 7 days | n | 1 | 3 |
|  | % (95% CI) | 0.2 (0.0;0.9) | 0.5 (0.1;1.5) |
| Use of traditional repellents over 7 days | n | 4 | 0 |
|  | % (95% CI) | 0.7 (0.2;1.7) | 0 (0.0;0.6) |
| Use of none of the above over 7 days | n | 598 | 577 |
|  | % (95% CI) | 99.0 (97.9;99.6) | 96.3 (94.5;97.7) |
| Use of IRS in past 12 months | n | 0 | 0 |
|  | % (95% CI) | 0 (0.0;0.6) | 0 (0.0;0.6) |
| **Kintampo, GH** |  | **N=600** | **N=600** |
| Use of mosquito coils over 7 days | n | 84 | 30 |
|  | % (95% CI) | 14.0 (11.3;17.0) | 5.0 (3.4;7.1) |
| Use of insecticide sprays over 7 days | n | 25 | 24 |
|  | % (95% CI) | 4.2 (2.7;6.1) | 4.0 (2.6;5.9) |
| Use of commercial repellents over 7 days | n | 0 | 0 |
|  | % (95% CI) | 0 (0.0;0.6) | 0 (0.0;0.6) |
| Use of traditional repellents over 7 days | n | 1 | 0 |
|  | % (95% CI) | 0.2 (0.0;0.9) | 0 (0.0;0.6) |
| Use of none of the above over 7 days | n | 490 | 560 |
|  | % (95% CI) | 81.7 (78.3;84.7) | 93.3 (91.0;95.2) |
| Use of IRS in past 12 months | n | 2 | 0 |
|  | % (95% CI) | 0.3 (0.0;1.2) | 0 (0.0;0.6) |
| **Kombewa, KE** |  | **N=599** | **N=599** |
| Use of mosquito coils over 7 days | n | 23 | 7 |
|  | % (95% CI) | 3.8 (2.4;5.7) | 1.2 (0.5;2.4) |
| Use of insecticide sprays over 7 days | n | 5 | 0 |
|  | % (95% CI) | 0.8 (0.3;1.9) | 0 (0.0;0.6) |
| Use of commercial repellents over 7 days | n | 1 | 2 |
|  | % (95% CI) | 0.2 (0.0;0.9) | 0.3 (0.0;1.2) |
| Use of traditional repellents over 7 days | n | 2 | 0 |
|  | % (95% CI) | 0.3 (0.0;1.2) | 0 (0.0;0.6) |
| Use of none of the above over 7 days | n | 568 | 590 |
|  | % (95% CI) | 94.8 (92.7;96.5) | 98.5 (97.2;99.3) |
| Use of IRS in past 12 months | n | 0 | 0 |
|  | % (95% CI) | 0 (0.0;0.6) | 0 (0.0;0.6) |
| **KeurSocé, SN** |  | **N=600** | **N=600** |
| Use of mosquito coils over 7 days | n | 8 | 4 |
|  | % (95% CI) | 1.3 (0.6;2.6) | 0.7 (0.2;1.7) |
| Use of insecticide sprays over 7 days | n | 5 | 0 |
|  | % (95% CI) | 0.8 (0.3;1.9) | 0 (0.0;0.6) |
| Use of commercial repellents over 7 days | n | 1 | 1 |
|  | % (95% CI) | 0.2 (0.0;0.9) | 0.2 (0.0;0.9) |
| Use of traditional repellents over 7 days | n | 11 | 81 |
|  | % (95% CI) | 1.8 (0.9;3.3) | 13.5 (10.9;16.5) |
| Use of none of the above over 7 days | n | 581 | 514 |
|  | % (95% CI) | 96.8 (95.1;98.1) | 85.7 (82.6;88.4) |
| Use of IRS in past 12 months | n | 286 | 0 |
|  | % (95% CI) | 47.7 (43.6;51.7) | 0 (0.0;0.6) |
| **Niakhar, SN** |  | **N=598** | **N=601** |
| Use of mosquito coils over 7 days | n | 13 | 11 |
|  | % (95% CI) | 2.2 (1.2;3.7) | 1.8 (0.9;3.3) |
| Use of insecticide sprays over 7 days | n | 3 | 4 |
|  | % (95% CI) | 0.5 (0.1;1.5) | 0.7 (0.2;1.7) |
| Use of commercial repellents over 7 days | n | 44 | 0 |
|  | % (95% CI) | 7.4 (5.4;9.8) | 0 (0.0;0.6) |
| Use of traditional repellents over 7 days | n | 102 | 22 |
|  | % (95% CI) | 17.1 (14.1;20.3) | 3.7 (2.3;5.5) |
| Use of none of the above over 7 days | n | 477 | 565 |
|  | % (95% CI) | 79.8 (76.3;82.9) | 94.0 (91.8;95.8) |
| Use of IRS in past 12 months | n | 12 | 28 |
|  | % (95% CI) | 2.0 (1.0;3.5) | 4.7 (3.1;6.7) |
| **Korogwe, TZ** |  | **N=601** | **N=600** |
| Use of mosquito coils over 7 days | n | 8 | 26 |
|  | % (95% CI) | 1.3 (0.6;2.6) | 4.3 (2.8;6.3) |
| Use of insecticide sprays over 7 days | n | 0 | 20 |
|  | % (95% CI) | 0 (0.0;0.6) | 3.3 (2.0;5.1) |
| Use of commercial repellents over 7 days | n | 0 | 2 |
|  | % (95% CI) | 0 (0.0;0.6) | 0.3 (0.0;1.2) |
| Use of traditional repellents over 7 days | n | 1 | 1 |
|  | % (95% CI) | 0.2 (0.0;0.9) | 0.2 (0.0;0.9) |
| Use of none of the above over 7 days | n | 592 | 552 |
|  | % (95% CI) | 98.5 (97.2;99.3) | 92.0 (89.5;94.0) |
| Use of IRS in past 12 months | n | 0 | 0 |
|  | % (95% CI) | 0 (0.0;0.6) | 0 (0.0;0.6) |
| **Overall** |  | **N=4,208** | **N=4,199** |
| Use of mosquito coils over 7 days | n | 246 | 305 |
|  | % (95% CI) | 5.8 (5.2;6.6) | 7.3 (6.5;8.1) |
| Use of insecticide sprays over 7 days | n | 52 | 65 |
|  | % (95% CI) | 1.2 (0.9;1.6) | 1.5 (1.2;2.0) |
| Use of commercial repellents over 7 days | n | 56 | 22 |
|  | % (95% CI) | 1.3 (1.0;1.7) | 0.5 (0.3;0.8) |
| Use of traditional repellents over 7 days | n | 122 | 106 |
|  | % (95% CI) | 2.9 (2.4;3.5) | 2.5 (2.1;3.0) |
| Use of none of the above over 7 days | n | 3,779 | 3,722 |
|  | % (95% CI) | 89.8 (88.9;90.7) | 88.6 (87.6;89.6) |
| Use of IRS in past 12 months | n | 300 | 42 |
|  | % (95% CI) | 7.1 (6.4;7.9) | 1.0 (0.7;1.3) |

N = total number of individuals overall or per site.

n = number of individuals in a given category.

95% CI = Exact 95% confidence limits.

IRS = Indoor Residual Spraying, application of a residual insecticide to internal walls and ceilings of housing structures.[^4^](#_ENREF_4)

BF = Burkina Faso; GH = Ghana; KE = Kenya; SN = Senegal; TZ = Tanzania.

## Care-seeking behaviours

SupplementalTable 8 Number of individualspresenting with fever reported in the last 24 hours and measured at visit by study site,*P. falciparum* infection status and survey

| **Study site** |  | ***Pf* infected** | | ***Pf* not infected** | | **Total** | |
| --- | --- | --- | --- | --- | --- | --- | --- |
|  |  | **Survey 1** | **Survey 2** | **Survey 1** | **Survey 2** | **Survey 1** | **Survey 2** |
| **Nouna, BF** |  | **N=403** | **N=488** | **N=203** | **N=112** | **N=606** | **N=600** |
| Fever in the last 24 hours* | n | 138 | 120 | 38 | 29 | 176 | 149 |
|  | % (95% CI) | 34.2 (29.6;39.1) | 24.6 (20.8;28.7) | 18.7 (13.6;24.8) | 25.9 (18.1;35.0) | 29.0 (25.5;32.8) | 24.8 (21.4;28.5) |
| Fever at visit** | n | 29 | 32 | 3 | 5 | 32 | 37 |
|  | % (95% CI) | 7.2 (4.9;10.2) | 6.6 (4.5;9.1) | 1.5 (0.3;4.3) | 4.5 (1.5;10.1) | 5.3 (3.6;7.4) | 6.2 (4.4;8.4) |
| **Saponé, BF** |  | **N=299** | **N=316** | **N=305** | **N=283** | **N=604** | **N=599** |
| Fever in the last 24 hours* | n | 15 | 32 | 7 | 15 | 22 | 47 |
|  | % (95% CI) | 5.0 (2.8;8.1) | 10.1 (7.0;14.0) | 2.3 (0.9;4.7) | 5.3 (3.0;8.6) | 3.6 (2.3;5.5) | 7.8 (5.8;10.3) |
| Fever at visit** | n | 13 | 25 | 8 | 8 | 21 | 33 |
|  | % (95% CI) | 4.4 (2.3;7.3) | 7.9 (5.2;11.5) | 2.6 (1.1;5.1) | 2.8 (1.2;5.5) | 3.5 (2.2;5.3) | 5.5 (3.8;7.7) |
| **Kintampo, GH** |  | **N=214** | **N=212** | **N=386** | **N=388** | **N=600** | **N=600** |
| Fever in the last 24 hours* | n | 102 | 105 | 83 | 142 | 185 | 247 |
|  | % (95% CI) | 47.7 (40.8;54.6) | 49.5 (42.6;56.5) | 21.5 (17.5;25.9) | 36.6 (31.8;41.6) | 30.8 (27.2;34.7) | 41.2 (37.2;45.2) |
| Fever at visit** | n | 22 | 22 | 11 | 8 | 33 | 30 |
|  | % (95% CI) | 10.3 (6.6;15.2) | 10.4 (6.6;15.3) | 2.8 (1.4;5.0) | 2.1 (0.9;4.0) | 5.5 (3.8;7.6) | 5.0 (3.4;7.1) |
| **Kombewa, KE** |  | **N=196** | **N=189** | **N=403** | **N=410** | **N=599** | **N=599** |
| Fever in the last 24 hours* | n | 127 | 146 | 255 | 283 | 382 | 429 |
|  | % (95% CI) | 64.8 (57.7;71.5) | 77.2 (70.6;83.0) | 63.3 (58.4;68.0) | 69.0 (64.3;73.5) | 63.8 (59.8;67.6) | 71.6 (67.8;75.2) |
| Fever at visit** | n | 18 | 15 | 14 | 4 | 32 | 19 |
|  | % (95% CI) | 9.2 (5.5;14.1) | 7.9 (4.5;12.8) | 3.5 (1.9;5.8) | 1.0 (0.3;2.5) | 5.3 (3.7;7.5) | 3.2 (1.9;4.9) |
| **KeurSocé, SN** |  | **N=3** | **N=6** | **N=597** | **N=594** | **N=600** | **N=600** |
| Fever in the last 24 hours* | n | 0 | 3 | 37 | 15 | 37 | 18 |
|  | % (95% CI) | 0 (0.0;70.8) | 50.0 (11.8;88.2) | 6.2 (4.4;8.4) | 2.5 (1.4;4.1) | 6.2 (4.4;8.4) | 3.0 (1.8;4.7) |
| Fever at visit** | n | 1 | 2 | 42 | 62 | 43 | 64 |
|  | % (95% CI) | 33.3 (0.8;90.6) | 33.3 (4.3;77.7) | 7.0 (5.1;9.4) | 10.4 (8.1;13.2) | 7.2 (5.2;9.5) | 10.7 (8.3;13.4) |
| **Niakhar, SN** |  | **N=9** | **N=3** | **N=589** | **N=598** | **N=598** | **N=601** |
| Fever in the last 24 hours* | n | 4 | 1 | 131 | 141 | 135 | 142 |
|  | % (95% CI) | 44.4 (13.7;78.8) | 33.3 (0.8;90.6) | 22.2 (18.9;25.8) | 23.6 (20.2;27.2) | 22.6 (19.3;26.1) | 23.6 (20.3;27.2) |
| Fever at visit** | n | 1 | 1 | 14 | 10 | 15 | 11 |
|  | % (95% CI) | 11.1 (0.3;48.2) | 33.3 (0.8;90.6) | 2.4 (1.3;4.0) | 1.7 (0.8;3.1) | 2.5 (1.4;4.1) | 1.8 (0.9;3.3) |
| **Korogwe, TZ** |  | **N=63** | **N=18** | **N=538** | **N=582** | **N=601** | **N=600** |
| Fever in the last 24 hours* | n | 36 | 6 | 87 | 44 | 123 | 50 |
|  | % (95% CI) | 57.1 (44.0;69.5) | 33.3 (13.3;59.0) | 16.2 (13.2;19.6) | 7.6 (5.5;10.0) | 20.5 (17.3;23.9) | 8.3 (6.2;10.8) |
| Fever at visit** | n | 24 | 1 | 15 | 5 | 39 | 6 |
|  | % (95% CI) | 38.1 (26.1;51.2) | 5.6 (0.1;27.3) | 2.8 (1.6;4.6) | 0.9 (0.3;2.0) | 6.5 (4.7;8.8) | 1.0 (0.4;2.2) |
| **Overall** |  | **N=1,187** | **N=1,232** | **N=3,021** | **N=2,967** | **N=4,208** | **N=4,199** |
| Fever in the last 24 hours* | n | 422 | 413 | 638 | 669 | 1,060 | 1,082 |
|  | % (95% CI) | 35.6 (32.8;38.4) | 33.5 (30.9;36.2) | 21.1 (19.7;22.6) | 22.5 (21.1;24.1) | 25.2 (23.9;26.5) | 25.8 (24.5;27.1) |
| Fever at visit** | n | 108 | 98 | 107 | 102 | 215 | 200 |
|  | % (95% CI) | 9.1 (7.5;10.9) | 8.0 (6.5;9.6) | 3.5 (2.9;4.3) | 3.4 (2.8;4.2) | 5.1 (4.5;5.8) | 4.8 (4.1;5.5) |

*Pf*infected = Individuals infected with *P. falciparum*parasitemia measured by microscopy.

*Pf* not infected = Individuals not infected with *P. falciparum*parasitemia measured by microscopy.

N = total number of individualsoverall or per site.

n = number of individuals in a given category.

95%CI = exact 95% confidence limits.

*Fever in the 24h prior to the visit reported during the visit.

**Temperature recorded at visit after axillary conversion ≥ 37.5°C.

BF = Burkina Faso; GH = Ghana; KE = Kenya; SN= Senegal; TZ = Tanzania.

Supplemental Table 9 Number of individuals presenting with feverreported in the last 24 hours and measured at visit by study site,parasite density measured by microscopyand survey

| **Study site** | **Parasitemia** | **Negative** | | **Low** | | **Medium** | | **High** | | **Very high** | |
| --- | --- | --- | --- | --- | --- | --- | --- | --- | --- | --- | --- |
|  |  | **Survey 1** | **Survey 2** | **Survey 1** | **Survey 2** | **Survey 1** | **Survey 2** | **Survey 1** | **Survey 2** | **Survey 1** | **Survey 2** |
| **Nouna, BF** |  | **N=203** | **N=112** | **N=198** | **N=307** | **N=105** | **N=100** | **N=48** | **N=39** | **N=52** | **N=42** |
| Fever in the last 24 hours* | n | 38 | 29 | 44 | 67 | 39 | 18 | 24 | 13 | 31 | 22 |
|  | %  (95% CI) | 18.7  (13.6;24.8) | 25.9  (18.1;35.0) | 22.2  (16.6;28.7) | 21.8  (17.3;26.9) | 37.1  (27.9;47.1) | 18.0  (11.0;26.9) | 50.0  (35.2;64.8) | 33.3  (19.1;50.2) | 59.6  (45.1;73.0) | 52.4  (36.4;68.0) |
| Fever at visit** | n | 3 | 5 | 5 | 12 | 3 | 3 | 7 | 3 | 14 | 14 |
|  | %  (95% CI) | 1.5  (0.3;4.3) | 4.5  (1.5;10.1) | 2.5  (0.8;5.8) | 3.9  (2.0;6.7) | 2.9  (0.6;8.1) | 3.0  (0.6;8.5) | 14.6  (6.1;27.8) | 7.7  (1.6;20.9) | 26.9  (15.6;41.0) | 33.3  (19.6;49.5) |
| **Saponé, BF** |  | **N=305** | **N=283** | **N=167** | **N=151** | **N=81** | **N=93** | **N=32** | **N=31** | **N=19** | **N=41** |
| Fever in the last 24 hours* | n | 7 | 15 | 6 | 6 | 5 | 11 | 0 | 4 | 4 | 11 |
|  | %  (95% CI) | 2.3  (0.9;4.7) | 5.3  (3.0;8.6) | 3.6  (1.3;7.7) | 4.0  (1.5;8.4) | 6.2  (2.0;13.8) | 11.8  (6.1;20.2) | 0  (0.0;10.9) | 12.9  (3.6;29.8) | 21.1  (6.1;45.6) | 26.8  (14.2;42.9) |
| Fever at visit** | n | 8 | 8 | 4 | 4 | 3 | 7 | 2 | 4 | 4 | 10 |
|  | %  (95% CI) | 2.6  (1.1;5.1) | 2.8  (1.2;5.5) | 2.4  (0.7;6.1) | 2.6  (0.7;6.6) | 3.7  (0.8;10.4) | 7.5  (3.1;14.9) | 6.3  (0.8;20.8) | 12.9  (3.6;29.8) | 21.1  (6.1;45.6) | 24.4  (12.4;40.3) |
| **Kintampo, GH** |  | **N=386** | **N=388** | **N=123** | **N=118** | **N=39** | **N=38** | **N=16** | **N=23** | **N=36** | **N=33** |
| Fever in the last 24 hours* | n | 83 | 142 | 49 | 43 | 19 | 23 | 8 | 14 | 26 | 25 |
|  | %  (95% CI) | 21.5  (17.5;25.9) | 36.6  (31.8;41.6) | 39.8  (31.1;49.1) | 36.4  (27.8;45.8) | 48.7  (32.4; 65.2) | 60.5  (43.4;76.0) | 50.0  (24.7;75.3) | 60.9  (38.5;80.3) | 72.2  (54.8;85.8) | 75.8  (57.7;88.9) |
| Fever at visit** | n | 11 | 8 | 9 | 3 | 1 | 3 | 1 | 4 | 11 | 12 |
|  | %  (95% CI) | 2.8  (1.4;5.0) | 2.1  (0.9;4.0) | 7.3  (3.4;13.4) | 2.5  (0.5;7.3) | 2.6  (0.1;13.5) | 7.9  (1.7;21.4) | 6.3  (0.2;30.2) | 17.4  (5.0;38.8) | 30.6  (16.3;48.1) | 36.4  (20.4;54.9) |
| **Kombewa, KE** |  | **N=403** | **N=410** | **N=100** | **N=84** | **N=42** | **N=44** | **N=25** | **N=15** | **N=29** | **N=46** |
| Fever in the last 24 hours* | n | 255 | 283 | 64 | 61 | 21 | 33 | 16 | 13 | 26 | 39 |
|  | %  (95% CI) | 63.3  (58.4;68.0) | 69.0  (64.3;73.5) | 64.0  (53.8;73.4) | 72.6  (61.8;81.8) | 50.0  (34.2;65.8) | 75.0  (59.7;86.8) | 64.0  (42.5;82.0) | 86.7  (59.5;98.3) | 89.7  (72.6;97.8) | 84.8  (71.1;93.7) |
| Fever at visit** | n | 14 | 4 | 2 | 1 | 4 | 3 | 2 | 2 | 10 | 9 |
|  | %  (95% CI) | 3.5  (1.9;5.8) | 1.0  (0.3;2.5) | 2.0  (0.2;7.0) | 1.2  (0.0;6.5) | 9.5  (2.7;22.6) | 6.8  (1.4;18.7) | 8.0  (1.0;26.0) | 13.3  (1.7;40.5) | 34.5  (17.9;54.3) | 19.6  (9.4;33.9) |
| **KeurSocé, SN** |  | **N=597** | **N=594** | **N=2** | **N=2** | **-** | **N=3** | **N=1** | **-** | **-** | **N=1** |
| Fever in the last 24 hours* | n | 37 | 15 | 0 | 0 | - | 2 | 0 | - | - | 1 |
|  | %  (95% CI) | 6.2  (4.4;8.4) | 2.5  (1.4;4.1) | 0  (0.0;84.2) | 0  (0.0;84.2) | - | 66.7  (9.4;99.2) | 0  (0.0;97.5) | - | - | 100  (2.5;100) |
| Fever at visit** | n | 42 | 62 | 0 | 1 | - | 0 | 1 | - | - | 1 |
|  | %  (95% CI) | 7.0  (5.1;9.4) | 10.4  (8.1;13.2) | 0  (0.0;84.2) | 50.0  (1.3;98.7) | - | 0  (0.0;70.8) | 100  (2.5;100) | - | - | 100  (2.5;100) |
| **Niakhar, SN** |  | **N=589** | **N=598** | **N=6** | **N=1** | **N=1** | **-** | **N=1** | **N=1** | **N=1** | **N=1** |
| Fever in the last 24 hours* | n | 131 | 141 | 2 | 0 | 0 | - | 1 | 0 | 1 | 1 |
|  | %  (95% CI) | 22.2  (18.9;25.8) | 23.6  (20.2;27.2) | 33.3  (4.3;77.7) | 0  (0.0;97.5) | 0  (0.0;97.5) | - | 100  (2.5;100) | 0  (0.0;97.5) | 100  (2.5;100) | 100  (2.5;100) |
| Fever at visit** | n | 14 | 10 | 0 | 0 | 0 | - | 0 | 0 | 1 | 1 |
|  | %  (95% CI) | 2.4  (1.3;4.0) | 1.7  (0.8;3.1) | 0  (0.0;45.9) | 0  (0.0;97.5) | 0  (0.0;97.5) | - | 0  (0.0;97.5) | 0  (0.0;97.5) | 100  (2.5;100 | 100  (2.5;100) |
| **Korogwe, TZ** |  | **N=538** | **N=582** | **N=24** | **N=10** | **N=18** | **N=2** | **N=3** | **N=2** | **N=18** | **N=4** |
| Fever in the last 24 hours* | n | 87 | 44 | 9 | 3 | 10 | 1 | 2 | 1 | 15 | 1 |
|  | %  (95% CI) | 16.2  (13.2;19.6) | 7.6  (5.5;10.0) | 37.5  (18.8;59.4) | 30.0  (6.7;65.2) | 55.6  (30.8;78.5) | 50.0  (1.3;98.7) | 66.7  (9.4;99.2) | 50.0  (1.3;98.7) | 83.3  (58.6;96.4) | 25.0  (0.6;80.6) |
| Fever at visit** | n | 15 | 5 | 3 | 1 | 6 | 0 | 1 | 0 | 14 | 0 |
|  | %  (95% CI) | 2.8  (1.6;4.6) | 0.9  (0.3;2.0) | 12.5  (2.7;32.4) | 10.0  (0.3;44.5) | 33.3  (13.3;59.0) | 0  (0.0;84.2) | 33.3  (0.8;90.6) | 0  (0.0;84.2) | 77.8  (52.4;93.6) | 0  (0.0;60.2) |
| **Overall** |  | **N=3,021** | **N=2,967** | **N=620** | **N=673** | **N=286** | **N=280** | **N=126** | **N=111** | **N=155** | **N=168** |
| Fever in the last 24 hours* | n | 638 | 669 | 174 | 180 | 94 | 88 | 51 | 45 | 103 | 100 |
|  | %  (95% CI) | 21.1  (19.7;22.6) | 22.5  (21.1;24.1) | 28.1  (24.6;31.8) | 26.7  (23.4;30.3) | 32.9  (27.5;38.6) | 31.4  (26.0;37.2) | 40.5  (31.8;49.6) | 40.5  (31.3;50.3) | 66.5  (58.4;73.8) | 59.5  (51.7;67.0) |
| Fever at visit** | n | 107 | 102 | 23 | 22 | 17 | 16 | 14 | 13 | 54 | 47 |
|  | %  (95% CI) | 3.5  (2.9;4.3) | 3.4  (2.8;4.2) | 3.7  (2.4;5.5) | 3.3  (2.1;4.9) | 5.9  (3.5;9.3) | 5.7  (3.3;9.1) | 11.1  (6.2;17.9) | 11.7  (6.4;19.2) | 34.8  (27.4;42.9) | 28.0  (21.3;35.4) |

Low = <2,500 parasites/ μL; Medium = 2,500 – 9,999 parasites/ μL; High 10,000 – 19,999 parasites/ μL; Very high ≥ 20,000 parasites/ μL.

N= total number of individualsoverall or per site.

n= number of individuals in a given category.

95%CI = Exact 95% confidence limits.

*Fever in the 24h prior to the visit reported during the visit.

**Temperature recorded at visit after axillary conversion ≥ 37.5°C.

BF = Burkina Faso; GH = Ghana; KE = Kenya; SN = Senegal; TZ = Tanzania.

Supplemental Table 10 Number of individualshaving sought treatment for malaria or fever in thepast 14 days and individuals hospitalized for malaria in the last 3 months by study site, *P. falciparum* infection status and survey

| **Study site** |  | ***Pf* infected** | | ***Pf* not infected** | | **Total** | |
| --- | --- | --- | --- | --- | --- | --- | --- |
|  |  | **Survey 1** | **Survey 2** | **Survey 1** | **Survey 2** | **Survey 1** | **Survey 2** |
| **Nouna, BF** |  | **N=403** | **N=488** | **N=203** | **N=112** | **N=606** | **N=600** |
| Seeking malaria or fever treatment in past 14 days | n | 132 | 125 | 70 | 33 | 202 | 158 |
|  | % (95% CI) | 32.8 (28.2;37.6) | 25.6 (21.8;29.7) | 34.5 (28.0;41.5) | 29.5 (21.2;38.8) | 33.3 (29.6;37.2) | 26.3 (22.8;30.1) |
| Malaria hospitalization in the last 3 months | n | 7 | 12 | 5 | 3 | 12 | 15 |
|  | % (95% CI) | 1.7 (0.7;3.5) | 2.5 (1.3;4.3) | 2.5 (0.8;5.7) | 2.7 (0.6;7.6) | 2.0 (1.0;3.4) | 2.5 (1.4;4.1) |
| **Saponé, BF** |  | **N=299** | **N=316** | **N=305** | **N=283** | **N=604** | **N=599** |
| Seeking malaria or fever treatment in past 14 days | n | 12 | 21 | 25 | 25 | 37 | 46 |
|  | % (95% CI) | 4.0 (2.1;6.9) | 6.6 (4.2;10.0) | 8.2 (5.4;11.9) | 8.8 (5.8;12.8) | 6.1 (4.3;8.3) | 7.7 (5.7;10.1) |
| Malaria hospitalization in the last 3 months | n | 2 | 4 | 4 | 5 | 6 | 9 |
|  | % (95% CI) | 0.7 (0.1;2.4) | 1.3 (0.3;3.2) | 1.3 (0.4;3.3) | 1.8 (0.6;4.1) | 1.0 (0.4;2.1) | 1.5 (0.7;2.8) |
| **Kintampo, GH** |  | **N=214** | **N=212** | **N=386** | **N=388** | **N=600** | **N=600** |
| Seeking malaria or fever treatment in past 14 days | n | 32 | 67 | 118 | 131 | 150 | 198 |
|  | % (95% CI) | 15.0 (10.5;20.4) | 31.6 (25.4;38.3) | 30.6 (26.0;35.4) | 33.8 (29.1;38.7) | 25.0 (21.6;28.7) | 33.0 (29.2;36.9) |
| Malaria hospitalization in the last 3 months | n | 9 | 17 | 33 | 38 | 42 | 55 |
|  | % (95% CI) | 4.2 (1.9;7.8) | 8.0 (4.7;12.5) | 8.5 (6.0;11.8) | 9.8 (7.0;13.2) | 7.0 (5.1;9.3) | 9.2 (7.0;11.8) |
| **Kombewa, KE** |  | **N=196** | **N=189** | **N=403** | **N=410** | **N=599** | **N=599** |
| Seeking malaria or fever treatment in past 14 days | n | 42 | 30 | 126 | 76 | 168 | 106 |
|  | % (95% CI) | 21.4 (15.9;27.8) | 15.9 (11.0;21.9) | 31.3 (26.8;36.0) | 18.5 (14.9;22.6) | 28.0 (24.5;31.8) | 17.7 (14.7;21.0) |
| Malaria hospitalization in the last 3 months | n | 11 | 9 | 21 | 24 | 32 | 33 |
|  | % (95% CI) | 5.6 (2.8;9.8) | 4.8 (2.2;8.8) | 5.2 (3.3;7.9) | 5.9 (3.8;8.6) | 5.3 (3.7;7.5) | 5.5 (3.8;7.7) |
| **KeurSocé, SN** |  | **N=3** | **N=6** | **N=597** | **N=594** | **N=600** | **N=600** |
| Seeking malaria or fever treatment in past 14 days | n | 0 | 0 | 0 | 0 | 0 | 0 |
|  | % (95% CI) | 0 (0.0;70.8) | 0 (0.0;45.9) | 0 (0.0;0.6) | 0 (0.0;0.6) | 0 (0.0;0.6) | 0 (0.0;0.6) |
| Malaria hospitalization in the last 3 months | n | 0 | 0 | 1 | 1 | 1 | 1 |
|  | % (95% CI) | 0 (0.0;70.8) | 0 (0.0;45.9) | 0.2 (0.0;0.9) | 0.2 (0.0;0.9) | 0.2 (0.0;0.9) | 0.2 (0.0;0.9) |
| **Niakhar, SN** |  | **N=9** | **N=3** | **N=589** | **N=598** | **N=598** | **N=601** |
| Seeking malaria or fever treatment in past 14 days | n | 0 | 0 | 6 | 0 | 6 | 0 |
|  | % (95% CI) | 0 (0.0;33.6) | 0 (0.0;70.8) | 1.0 (0.4;2.2) | 0 (0.0;0.6) | 1.0 (0.4;2.2) | 0 (0.0;0.6) |
| Malaria hospitalization in the last 3 months | n | 0 | 0 | 0 | 0 | 0 | 0 |
|  | % (95% CI) | 0 (0.0;33.6) | 0 (0.0;70.8) | 0 (0.0;0.6) | 0 (0.0;0.6) | 0 (0.0;0.6) | 0 (0.0;0.6) |
| **Korogwe, TZ** |  | **N=63** | **N=18** | **N=538** | **N=582** | **N=601** | **N=600** |
| Seeking malaria or fever treatment in past 14 days | n | 20 | 4 | 79 | 25 | 99 | 29 |
|  | % (95% CI) | 31.7 (20.6;44.7) | 22.2 (6.4;47.6) | 14.7 (11.8;18.0) | 4.3 (2.8;6.3) | 16.5 (13.6;19.7) | 4.8 (3.3;6.9) |
| Malaria hospitalization in the last 3 months | n | 4 | 0 | 12 | 6 | 16 | 6 |
|  | % (95% CI) | 6.3 (1.8;15.5) | 0 (0.0;18.5) | 2.2 (1.2;3.9) | 1.0 (0.4;2.2) | 2.7 (1.5;4.3) | 1.0 (0.4;2.2) |
| **Overall** |  | **N=1,187** | **N=1,232** | **N=3,021** | **N=2,967** | **N=4,208** | **N=4,199** |
| Seeking malaria or fever treatment in past 14 days | n | 238 | 247 | 424 | 290 | 662 | 537 |
|  | % (95% CI) | 20.1 (17.8;22.4) | 20.0 (17.8;22.4) | 14.0 (12.8;15.3) | 9.8 (8.7;10.9) | 15.7 (14.6;16.9) | 12.8 (11.8;13.8) |
| Malaria hospitalization in the last 3 months | n | 33 | 42 | 76 | 77 | 109 | 119 |
|  | % (95% CI) | 2.8 (1.9;3.9) | 3.4 (2.5;4.6) | 2.5 (2.0;3.1) | 2.6 (2.1;3.2) | 2.6 (2.1;3.1) | 2.8 (2.4;3.4) |

*Pf*infected = Individuals infected with *P. falciparum*parasitemia measured by microscopy.

*Pf* not infected = Individuals not infected with *P. falciparum*parasitemia measured by microscopy.

N = total number of individuals overall or per site.

n = number of individualsin a given category.

95% CI = Exact 95% confidence limits.

BF = Burkina Faso; GH = Ghana; KE = Kenya; SN = Senegal; TZ = Tanzania.

## *P. falciparum* infection risk factors analysis

Supplemental Table 11 Risk factors of being infected with *P. falciparum* (as assessed by microscopy)derived from the fitted logistic regression model with study site as cluster (Survey 1)

| **Characteristics** | **Category** | **Reference category** | **Odds ratio (OR)** | **95% CI of OR** |
| --- | --- | --- | --- | --- |
| Age (in years)* | Continuous |  | 1.141 | [1.048;1.242] |
| Antimalarial drugs consumed in the last 14 days | Yes | No | 0.614 | [0.376;1.002] |
| Antimalarial or any other medication within 14 days | Yes | No | 1.033 | [0.772;1.383] |
| Main house construction material: Nets | Nets present on all windows | Nets not present | 0.729 | [0.631;0.842] |
|  | Nets present on some windows | Nets not present | 1.003 | [0.688;1.463] |
|  | Other | Nets not present | 1.130 | [1.019;1.252] |
| Main house construction material: Roof | Iron sheet | Grass/Palm | 0.888 | [0.753;1.046] |
|  | Tiles | Grass/Palm | 1.090 | [0.786;1.513] |
|  | Clay | Grass/Palm | 1.824 | [1.593;2.088] |
|  | Other | Grass/Palm | 1.175 | [1.063;1.300] |
| Main house construction material: Walls | Brick | Mud | 0.860 | [0.747;0.990] |
|  | Cement/Plaster | Mud | 0.811 | [0.685;0.961] |
|  | Cement /Paint | Mud | 0.842 | [0.638;1.113] |
|  | Clay | Mud | 0.612 | [0.214;1.752] |
|  | Other | Mud | 0.622 | [0.476;0.812] |
| Main house construction material: Windows/eaves | Closed | Open | 0.984 | [0.845;1.144] |
|  | No Windows | Open | 0.959 | [0.810;1.137] |
|  | Partially open | Open | 0.987 | [0.794;1.228] |
|  | Other | Open | 1.374 | [1.234;1.530] |
| Number of holes | < 5 | ≥5 | 0.921 | [0.712;1.191] |
|  | No bednet | ≥5 | 1.044 | [0.740;1.473] |
|  | No pierced bednet | ≥5 | 1.055 | [0.813;1.369] |
| Number of persons living in the same part of the house | 4-5 | ≤3 | 1.168 | [1.000;1.364] |
|  | >5 | ≤3 | 1.317 | [1.165;1.488] |
| Pierced/torn bednet | No bednet | No | 1.000 | [1.000;1.000] |
|  | Yes | No | 1.000 | [1.000;1.000] |
| Presence of electricity | Yes | No | 0.752 | [0.611;0.926] |
| Use of traditional repellents over 7 days | Yes | Missing/No | 0.994 | [0.907;1.088] |

Note: Odds ratios were adjusted by a cluster variable.

95% CI = Exact 95% confidence limits.

The fitted logistic regression model was only applied on significant variables produced by the backward selection.

The variables ‘Floor’, ‘number of persons enrolled into the study’ and ‘localisation’ were not included in the model due to convergence issue.

*Age was introduced in the model as a continuous variable. The corresponding OR is given for a 1 year increase of age.

Supplemental Table 12 Risk factors of being infected with *P. falciparum* (as assessed by microscopy)derived from the fitted logistic regression model with study site as cluster (Survey 2)

| **Characteristics** | **Category** | **Reference category** | **Odds ratio (OR)** | **95% CI of OR** |
| --- | --- | --- | --- | --- |
| Age (in years)* | Continuous |  | 1.087 | [1.018;1.161] |
| Antimalarial drug consumed in the past 14 days | Yes | No | 0.457 | [0.324;0.645] |
| Impregnated Bednet | No Bednet | No | 1.000 | [1.000;1.000] |
|  | Yes | No | 1.093 | [0.887;1.345] |
| Localization | Semi- Rural Area | Rural area | 0.741 | [0.419;1.308] |
|  | Urban area | Rural area | 0.185 | [0.109;0.314] |
| Main house construction material: Nets | Nets present on all windows | Nets not present | 0.787 | [0.660;0.939] |
|  | Nets present on some windows | Nets not present | 0.947 | [0.901;0.995] |
|  | Other | Nets not present | 1.007 | [0.886;1.143] |
| Main house construction material: Roof | Iron sheet | Grass/Palm | 0.953 | [0.818;1.111] |
|  | Tiles | Grass/Palm | 0.995 | [0.703;1.407] |
|  | Clay | Grass/Palm | 1.011 | [0.913;1.120] |
|  | Other | Grass/Palm | 1.236 | [0.786;1.943] |
| Main house construction material: Walls | Brick | Mud | 0.922 | [0.783;1.085] |
|  | Cement/Plaster | Mud | 0.866 | [0.757;0.992] |
|  | Cement /Paint | Mud | 0.990 | [0.853;1.148] |
|  | Clay | Mud | 1.197 | [1.120;1.278] |
|  | Other | Mud | 0.658 | [0.415;1.044] |
| Main source of drinking water | Closed water source^†^ | Open water source^††^ | 0.921 | [0.835;1.015] |
| Malaria or fever treatment sought for in the past 14 days | Yes | No | 1.227 | [1.047;1.439] |
| New net (less than 1 year) | No Bednet | No | 1.095 | [0.901;1.330] |
|  | Yes | No | 0.929 | [0.839;1.029] |
| Pierced/torn bednet | No Bednet | No | 1.000 | [1.000;1.000] |
|  | Yes | No | 0.912 | [0.798;1.042] |
| Presence of electricity | Yes | No | 0.885 | [0.803;0.976] |
| Use of traditional repellents over 7 days | Yes | Missing/No | 0.910 | [0.747;1.109] |
| No use of mosquito coils–insecticide sprays–commercial or traditional repellents over 7 days | Yes | Missing/No | 0.879 | [0.782;0.988] |

Note: Odds ratios (ORs) were adjusted by a cluster variable.

95% CI = Exact 95% confidence limits.

The fitted logistic regression model was only applied on significant variables produced by the backward selection.

The variables ‘Number of persons living in the same part of the house’, ‘Type of location’, ‘Floor’, ‘Windows/eaves’ and ‘Use of Insecticide sprays over 7 days’ were not included in the model due to convergence issue.

*Age was introduced in the model as a continuous variable. The corresponding OR is given for a 1 year increase of age.

^†^Closed water source (piped water, tube well, dug well, protected well).

^††^Open water source (unprotected well, spring water, rainwater, tanker truck, surface water).

# REFERENCES

1. World Health Organization. Basic Malaria Microscopy Part I: Learner's Guide. Available at <http://whqlibdoc.who.int/publications/1991/9241544309.pdf>.

2. Hermsen CC, Telgt DS, Linders EH, van de Locht LA, Eling WM, Mensink EJ, Sauerwein RW, 2001. Detection of Plasmodium falciparum malaria parasites in vivo by real-time quantitative PCR. Mol Biochem Parasitol. 118:247-251.

3. Schneider P, Schoone G, Schallig H, Verhage D, Telgt D, Eling W, Sauerwein R, 2004. Quantification of Plasmodium falciparum gametocytes in differential stages of development by quantitative nucleic acid sequence-based amplification. Mol Biochem Parasitol. 137:35-41.

4. World Health Organization. Indoor residual spraying: An operational manual for IRS for malaria transmission, control and elimination. Available at https://[www.who.int/malaria/publications/atoz/9789241508940/en/](http://www.who.int/malaria/publications/atoz/9789241508940/en/).
